# Mitigating Electrochemical Isolation in Ni‐Rich Layered Cathodes for Durable Solid‐State Batteries

**DOI:** 10.1002/advs.202518327

**Published:** 2026-01-28

**Authors:** Abhirup Bhadra, Maxime Brunisholz, Aditya Rawal, Jacob Otabil Bonsu, Tongjun Luo, Lars Thomsen, Wesley M. Dose, Dipan Kundu

**Affiliations:** ^1^ LBRI, School of Chemical Engineering UNSW Sydney Kensington New South Wales Australia; ^2^ MWAC UNSW Sydney Kensington New South Wales Australia; ^3^ School of Chemistry The University of Sydney Sydney New South Wales Australia; ^4^ Australian Synchrotron ANSTO Clayton Victoria Australia

**Keywords:** cathode‐electrolyte interphase, electrochemical isolation, interfacial stabilization, Ni‐rich NMC cathode, solid‐state batteries, sulfide solid electrolyte

## Abstract

Cathode–solid electrolyte (SE) interfacial instability poses a major challenge for achieving stable and high‐power operations in all‐solid‐state batteries, which promise superior energy density, thermal stability, and safety over the current Li‐ion technology. For technologically important Ni‐rich NMCs (LiNi_x_Mn_y_Co_z_O_2_ or NMCxyz; x/y/z: Ni/Mn/Co stoichiometry) paired with sulfide SEs, redox‐mediated instability of the SE is often blamed for rapid cathode deterioration. Here, in‐depth spectroscopic and electrochemical analyses of Ni‐rich NMCs with a promising sulfide SE reveal hitherto unrecognized electrochemical isolation of active NMC particles driven by rapid interfacial degradations, sparking accelerated capacity fading and poor thermal stability. Introducing a functionalized conductive carbon into the cathode suppresses sulfide SE degradation into reactive polysulfides that drive NMC deterioration. Consequently, NMC622 and NMC811‐based cells display high active material utilization, enhanced stability, attractive rate capability and thermal resilience – illustrated by 1C (1C: 160 mA g^−1^) capacity of ∼150 mAh g^−1^, 5C rate retention of 95% after 500 cycles with high active loading (≥12 mg cm^−2^), and an average Coulombic efficiency of 99.8% even for high‐temperature cycling. This study uncovers a critical performance degradation pathway in a key cathode‐SE pairing and presents a scalable strategy for its in situ regulation, enabling significant performance gains.

## Graphical abstract

Electrochemical isolation of Ni‐rich layered cathodes in sulfide solid‐state batteries is uncovered as a previously unrecognized but critical performance‐limiting pathway. This study elucidates its origin and impact and introduces a scalable mitigation strategy using oxygen‐functionalized conductive carbon. The approach enables high‐rate capability, thermal resilience, and long‐term cycling stability, advancing the practical deployment of Ni‐rich cathodes in solid‐state systems.

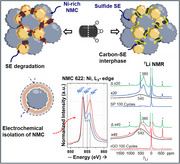

## 1 Introduction

Innate safety and the potential for high energy density make all‐solid‐state batteries (ASSBs) a promising alternative to conventional Li‐ion batteries for energy‐demanding applications. While remarkable progress has been made over the past years, several pressing challenges remain toward enabling prolonged cycling stability, high power characteristics, scalable processibility and manufacturing, and low‐pressure operation, amongst others. A key to addressing some of these technical challenges is achieving stable and seamless ionic/electronic contacts across solid‐solid interfaces. Sulfide solid electrolytes (SEs) with attractive high ionic conductivity and attributes like room temperature formability, originating from superior elasticity and softer grain boundaries [1, 2], can overcome poor interfacial contact issues faced by oxide‐type SEs and enable appealing electrochemical performance.

Yet, the potential integration of sulfide SEs into ASSBs has been marred by their rather narrow electrochemical stability window [3, 4, 5, 6], leading to interfacial degradations at both anode and cathode interfaces, which impede charge transport and negatively impact obtainable capacity and cycling stability [7, 8]. Such interfacial issues pose a major challenge toward developing stable high‐energy and high‐power ASSB chemistries employing high voltage and high capacity layered oxide cathode active materials, e.g., high nickel‐containing Li(Ni_x_Mn_y_Co_z_)O_2_ (or NMCxyz where x/y/z refers to the transition metal stoichiometry). Conductive carbon additives, typically added to the electrode composite to overcome the limited electronic conductivity of NMC materials (NMC111: 5.2 × 10^−8^ S cm^−^^1^, NMC 622: 1.6 × 10^−6^ S cm^−^^1^, NMC 811: 1.7 × 10^−5^ S cm^−^^1^) [9], is known to further compound NMC‐sulfide SE interfacial reaction kinetics [10, 11, 12, 13, 14], accelerating the electrochemical performance degradation. Our recent investigation [15] involving NMC111 confirmed that even modest carbon loadings can catalyze the oxidative decomposition of Li_6_PS_5_Cl into reactive polysulfides, which in turn trigger cathode surface degradation, underscoring the critical role of carbon in mediating interfacial instability in sulfide SE‐based ASSBs.

Several studies have indicated oxidative electrochemical degradation of sulfide electrolytes, with the formation of polysulfides, sulfates, and phosphates as the major degradation products at the cathode electrolyte interface [10, 11, 12, 13, 14, 16, 17, 18]. Although metal sulfides have been theoretically predicted as one of the NMC degradation products, it is yet to be experimentally verified [13, 19, 20, 21, 22]. A recent study involving near‐edge X‐ray absorption fine structure (NEXAFS) spectroscopy investigation of the NMC532 cathode composite with Li_6_PS_5_Cl at different degrees of chemical lithiation or state‐of‐charge (SOC) [23] revealed a preferential order—Ni^4+/3+^ > Mn^4+^/ Co^3+^ > Ni^2+^—of NMC transition metal reactivity toward Li_6_PS_5_Cl. However, the long‐term implications of such preferential susceptibility toward interfacial reactions were not probed.

In this context, the present study takes a deeper look into the interfacial stability of nickel‐rich NMC cathodes NMC622 and NMC811 in Li_6_PS_5_Cl SE‐based ASSBs. As revealed by in‐depth electrochemical and spectroscopic investigations, reactive polysulfides, arising from the redox‐mediated degradation of Li_6_PS_5_Cl, not only degrade the NMC to form thiosulfate, sulfate, and phosphate‐type insulating cathode electrolyte interphase (or CEI) but also result in electrochemical isolation of a considerable fraction of the active NMC particles. Here, the term ‘electrochemical isolation’ refers to the phenomenon where NMC particles become electronically or ionically disconnected from the ion and electron percolating network due to interfacial degradation, rendering them partially or fully inactive. Alongside, a new differential capacity (dQ/dV) feature emerges at lower potentials, reflecting the polarized but partial redox response from this isolated NMC fraction. Such a degradation manifests as a rapid deterioration of NMC622 and NMC811 ASSB cell capacity, even during room temperature cycling, which is, however, not observed for low‐Ni NMC111. Introducing a mildly oxygen‐functionalized conductive carbon in cathode composite formulation effectively shuts down the redox activity of Li_6_PS_5_Cl and, thus, its conversion to reactive polysulfides and associated NMC degradation. As a result, improved conductivity of the cathode composite, on the one hand, leads to enhanced NMC utilization and, therefore, high specific capacities and excellent current capability. Excellent NMC interfacial stability, on the other hand, renders attractive capacity retention over long‐term cycling and superior thermal stability. Unlike the coating of NMC particles with electronic insulators like Li_2_CO_3_, LiNbO_3_, Li_2_SiO_3_, etc. [20, 24, 25, 26, 27, 28, 29, 30, 31], which impedes electron/ion transport across NMC‐NMC, NMC‐carbon, and NMC‐SE interfaces, the in situ regulation of Li_6_PS_5_Cl reactivity through the surface‐engineered conductive carbon presents a highly scalable strategy with unparalleled performance potential.

## 2 Results and Discussion

### 2.1 Unique Electrochemical Evolution of Ni‐Rich NMCs in Li_6_PS_5_Cl SE‐Based Sulfide ASSBs

**FIGURE 1:**
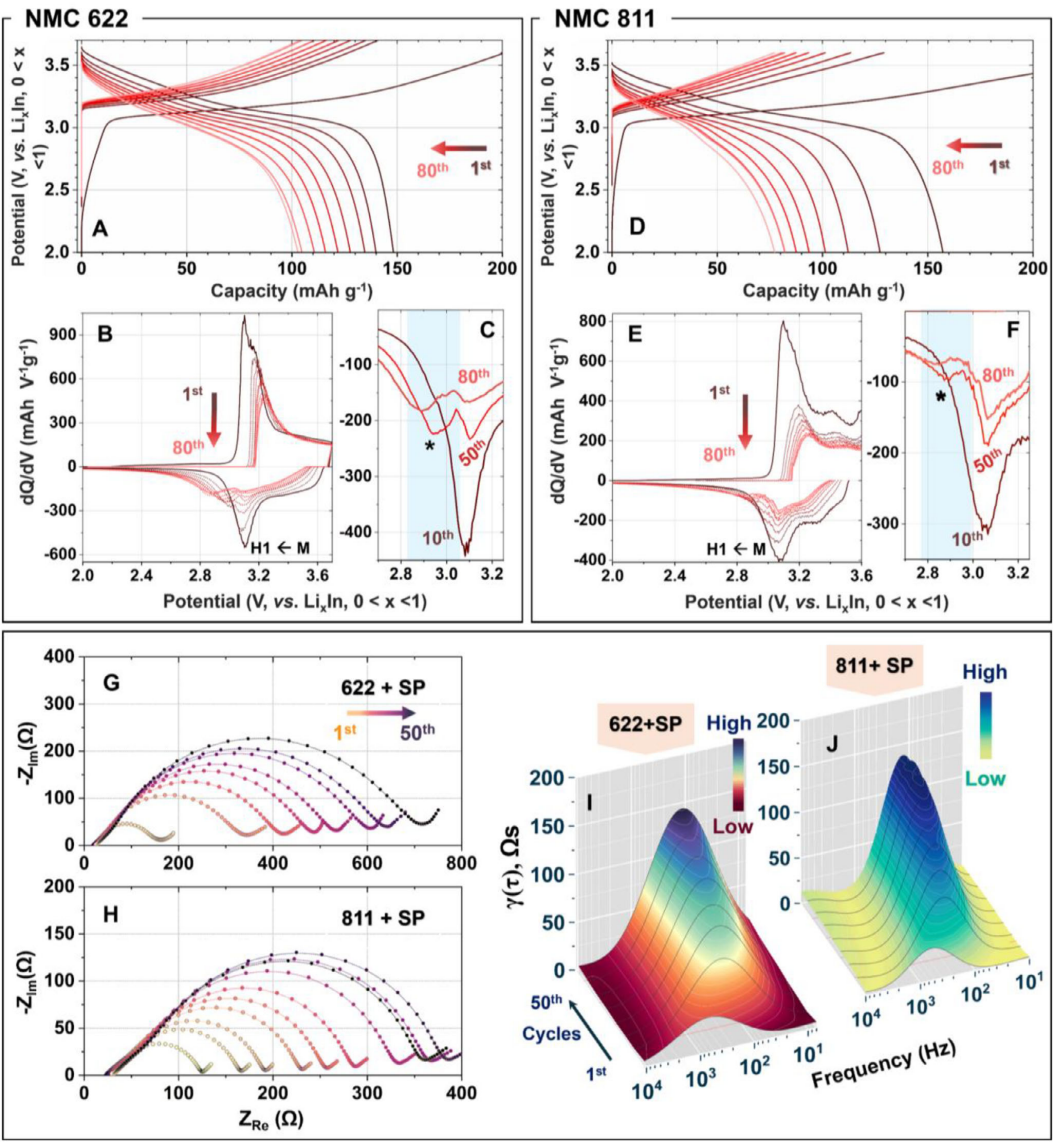
Room temperature electrochemistry indicates a unique Ni‐rich NMC—Li_6_PS_5_Cl interfacial trouble. (A) Evolution of the galvanostatic polarization profile for NMC622‐based ASSB cell and the corresponding (B,C) dQ/dV profile. (D) Evolution of the galvanostatic polarization profile for NMC811‐based ASSB cell and the corresponding (E,F) dQ/dV profile. The cells were cycled at the constant laboratory temperature (20 ± 2 °C) at a 0.2C rate, where 1C = 160 mAh g^−1^, within 2.0–3.7 V for NMC622 and within 2.0–3.6 V for NMC811 (vs. Li_x_In 0 < x < 1 anode). An active cathode loading of ≥12 mg cm^−2^ was applied in all cells unless otherwise specified. Here, SP stands for the conductive Super P carbon black. Nyquist impedance data for (G) NMC622 and (H) NMC811 cells and the corresponding distribution of relaxation time data for (I) NMC622 and (J) NMC811 present the impedance evolution with cycling. The impedance data shown was recorded in situ over 50 cycles after every 7^th^ cycle in the charged or 100% SOC state.

NMC622 and NMC811 were chosen as high nickel‐containing NMCs as they were readily available commercially. Commonly employed conductive carbon black Super P—denoted here as SP—was included in the cathode composite formulation to engage the majority of the NMC particles electronically, which is desired for an overall improved capacity and current capability (Figure S1). The employed NMC622 (MSE Supplies LLC) is composed of single‐crystalline particles with an average size of 2–5 µm, while NMC811 (MSE Supplies LLC) consists of polycrystalline secondary particles (∼5–13 µm) formed by the agglomeration of smaller primary crystallites (Figure S2). Figure 1 presents the room temperature (20 ± 2 °C, thermostated laboratory temperature) galvanostatic cycling behavior of the corresponding cells and the impedance evolution with cycling. The initial discharge capacity of the NMC622 cell, ∼147 mAh g^−1^, is somewhat moderate compared to reports involving nanoscale single‐crystalline particles [32, 33], which typically exhibit higher active material utilization due to shorter Li^+^ diffusion paths and more accessible surface area. Nevertheless, as revealed by galvanostatic polarization profiles in Figure 1A, the NMC622 cell displays a rapid decay in capacity with cycling, with the discharge capacity dropping to 83.5 mAh g^−1^ within the first 80 cycles. This drop is in stark contrast to the rather stable cycling of the corresponding NMC111 cell in the same voltage window (2–3.7 V vs. Li_0.5_In or ∼2.6–4.3 V vs. Li; Figure S3a) and points to the impact of the NMC Ni content on the cell degradation. The differential capacity or dQ/dV plot shown in Figure 1B,C provides further insights into the nature of the NMC622 cell electrochemical degradation. Upon prolonged cycling, the primary cathodic (reduction) dQ/dV peak corresponding to the monoclinic (M) → hexagonal (H1) phase transition [9, 34] appears with a shoulder at a slightly lower potential (∼2.85 V vs. Li_0.5_In; marked ^*^ in Figure 1C), representing a reaction pathway likely involving a secondary phase. While the corresponding dQ/dV signature is not apparent on the anodic (oxidative) half, its presence below the broad dQ/dV signature of NMC622 cannot be ruled out. Interestingly, this new reaction pathway, involving the secondary phase, is not observed for NMC111 (Figure S3b) during even the first 100 cycles.

The galvanostatic polarization profiles and the corresponding dQ/dV data for NMC811, as shown in Figure 1D–F, concur with the unique degradation pathway observed for NMC622. Notably, the NMC811 cell was probed under identical conditions, except in a reduced voltage window (2–3.6 V vs. Li_0.5_In), to avoid any interference from oxygen evolution accompanying H2 → H3 phase transformation at higher voltages (Figure S4 and associated discussion) [34]. Anyhow, the capacity fading is evidently quite aggressive here, with a rapid increase in the voltage polarization (Figure 1D). The dQ/dV plots in Figure 1E,F reveal an identical evolution of the dQ/dV signature as NMC622 with the appearance of the shoulder peak to M → H1 transformation peak during the cathodic cycle. However, the absence of the same for NMC111, which has an identical phase transformation as NMC622, and the absence of H2 → H3 or prominent M → H2 transformations [34], points to the Ni content in NMC as likely the key contributing factor in the observed degradation.

Electrochemical impedance spectroscopy (EIS), performed during galvanostatic cycling, sheds further light on the stability and evolution of the cathode‐electrolyte interface. Measurements were performed at both 100% SOC (fully charged) and 0% SOC or 100% DOD (fully discharged) conditions after allowing the cell to reach equilibrium by resting at OCV for 2 h. For NMC622, the 0% SOC Nyquist impedance data (Figure S5) does not offer any apparent evolution trend. NMC811, however, shows a significant increase in the mid/high‐frequency semicircle with cycling. As shown in Figure 1G,H for NMC622 and NMC811, respectively, the 100% SOC Nyquist data, on the other hand, show a steady increase in the semicircle. The slightly lower overall impedance for NMC811 compared to that for NMC622 can be ascribed to the intrinsically better electronic conductivity of NMC811 material and the corresponding cathode composite (Table S1 and Figure S6). Distribution of relaxation time or DRT analysis of the impedance data presented in Figure 1I,J, and Figure S7 unveils a clearer picture of different contributions and their evolution. The presence of two significant processes or interactions contributing toward the impedance build‐up is evident, as denoted by P_Cat‐MF_ and P_LF_ in Figure S7, which were assigned based on previous DRT analysis of NMC111 ASSB cells by our team and NMC 622 cells by Orue Mendizabal et al., [15, 35]. The contributions toward γ(τ) in the mid‐frequency region (10^4^ to 10^1^ in Hz scale) primarily correspond to the overall interfacial contribution encompassing both CEI (cathode particle‐SE) and CnEI (carbon‐SE interphase) components. As the P_LF_ region is primarily associated with the cathode interfacial charge transfer, 100% SOC or maximum de‐lithiated state data, which reduces the charge transfer contribution, explicitly highlights the impedance change in the P_Cat‐MF_ region from cathode interfacial degradations. The steady rise in the P_Cat‐MF_ region impedance with cycling for both NMC622 and NMC811 in Figure 1I,J highlights an increase in the overall resistive nature of the CEI, which contributes to increasing cell polarization and capacity fading.

It is worth noting that cycling NMC622 and NMC811 in LiPF_6_‐carbonate‐based electrolytes within an upper voltage limit of 4.3 V vs. graphite (equivalent of ∼3.8 V vs. Li_0.5_In) also shows pronounced capacity decay along with a significant CEI build‐up, but unlike here, no new dQ/dV signature is observed except for a progressive shift of the dQ/dV peaks with cycling [36]. Pushing the charging voltage further (>4.5 V vs. Li) in the liquid cell for Ni‐rich NMCs (typically for Ni content of ≥ 80% of the transition metals) leads to a myriad of correlated degradations, e.g., the formation of a surface reconstruction layer consisting of a reduced rock‐salt phase [37], partial structural transformation to a spinel phase [38], and interlayer NiO_2_ micrograin growth concurrent with a rising dQ/dV peak [39] with cycling, albeit at 4.15 V vs. Li (equivalent to ∼3.55 V vs. Li_0.5_In). Such degradations typically accompany repeated H2 → H3 transformations at high voltages [40]. Yet, the dQ/dV peak evolution observed here at around 2.85 V vs. Li_0.5_In (equivalent to ∼3.45 V vs. Li) for the SP‐based NMC622 (no H2 → H3 transformation) and NMC811 cathode cycling with sulfide solid electrolyte Li_6_PS_5_Cl is distinct and has not previously been reported for liquid or solid electrolyte systems [40]. Given the known reactivity of Ni^4+^ of charged Ni‐rich NMC with liquid electrolytes, producing gaseous and solid decomposition products like HF, CO_2_, CO, organic compounds, fluorine, and phosphorous derivatives, an aggravated reaction with reactive polysulfides, formed upon oxidative degradation of Li_6_PS_5_Cl (see below), appears plausible. The resulting electronically and ionically insulating CEI may progressively isolate a fraction of NMC particles from the electrochemical network. Such isolation could plausibly explain the emergence of a secondary dQ/dV feature at lower potentials, distinct from the bulk NMC response.

### 2.2 Functionalized Conductive Carbon Stabilizes Ni‐rich NMC Electrochemistry With Li_6_PS_5_Cl

**FIGURE 2:**
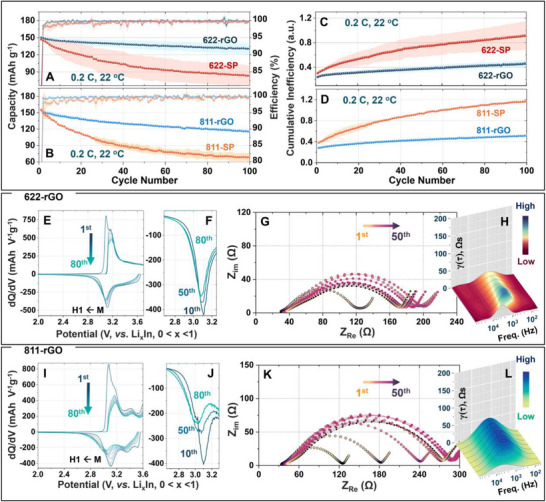
Functionalized conductive carbon stabilizes Ni‐rich NMC electrochemistry with Li_6_PS_5_Cl. Galvanostatic cyclability of the rGO‐based (A) NMC622 and (B) NMC811 ASSB cells with Li_6_PS_5_Cl solid electrolyte vis‐à‐vis the corresponding SP carbon‐based cells; (C,D) show the comparison of the corresponding cumulative inefficiency evolution as a function of cycling. Evolution of the dQ/dV profile as a function of cycling for the rGO‐based (E,F) NMC622 cell and (I,J) NMC811 cell and the corresponding (G,K) Nyquist impedance and (H,L) distribution of relaxation time (DRT) profile evolution. The cells were cycled at the constant laboratory temperature (22 °C) at a 0.2C rate with a Li_x_In (0 < x < 1) anode). The impedance data shown was recorded in situ over 50 cycles after every 7^th^ cycle in the charged or 100% SOC state.

Replacing SP carbon with equally conductive (∼1–2 S cm^−1^, Table S1 and Figure S6) but mildly oxygen functionalized reduced graphene oxide (2 weight% oxygen content with predominantly ether (C─O─C) and carbonyl (C═O) functionalities; Figure S8) upturns the electrochemical behavior of the ASSB cells. Figure 2A,B compare the galvanostatic cyclability for rGO composite cathodes against the SP‐containing ones for which the data have been presented in Figure 1. While the starting capacities are comparable, against the rapid decline of capacity for the SP cells, rGO cells display retention of 87% and 75% for NMC622 and 811, respectively. Although the Coulombic efficiency data, which are ∼99.8% for both rGO cells compared to 99.4% and 99.2% (Table S3) for the SP‐based NMC622 and NMC811 cells, respectively, point to a greater extent of degradation in the SP cells, comparison of the cumulative inefficiency data (Figure 2C,D) magnifies the difference. The cumulative inefficiency, which parameterizes the degradation progress in terms of cumulative coulomb loss (see methods section for details) [15], increases much more steeply for the SP cells with cycling, highlighting the heightened interfacial degradations. Unlike SP, rGO in the cathode composite steadies the galvanostatic polarization significantly (Figure S9), and inhibits the secondary phase evolution, as is evident from the dQ/dV data shown in Figure 2E,F,I, and J. Although the dQ/dV peaks show a small shift due to the slight increase in polarization with cycling, as apparent from the Nyquist impedance evolution with cycling (Figure 2G,K; Figure S10), there is no sign of the shoulder peak to M → H1 transition as observed for the SP cathodes during the cathodic cycle. Furthermore, much stable Nyquist impedance data and the corresponding DRT profile (Figure 2H,L; Figure S11) evolution as a function of cycling further supports the inhibition of accelerated cathode interfacial degradations, which is otherwise apparent for the SP cells (see above). The slight decrease in γ (τ) with increasing cycle number for rGO cells, particularly for NMC622, suggests cell conditioning and an improvement in the electronic and ionic contact in the cathode composite with cycling. The NMC811 cell, however, displays a γ (τ) rise with cycling, consistent with a larger shift in the dQ/dV profile and a higher capacity decay than the NMC622 cell. Nevertheless, the impact of rGO in the cathode composite can be rationalized in terms of our previously established observation for NMC111 and sulfide SE‐based ASSBs. We found that oxygen‐containing (reactive) surface functional groups of conductive carbon lead to an electrically insulating interphase at the carbon‐SE interface (referred to as CnEI, contrary to CEI for the NMC‐SE interphase) [15], which subdues the well‐acknowledged redox‐mediated degradation of Li_6_PS_5_Cl into reactive polysulfides, which promotes NMC degradations. It is important to note that the formation of reactive polysulfides is not confined to the carbon‐SE interface; rather, these species originate from the electrochemical oxidation of Li_6_PS_5_Cl and can form across the SE surface layer, enabling subsequent interaction with active NMC particles.

### 2.3 X‐Ray Photoelectron Spectroscopy (XPS) Investigation of the CEI Evolution

**FIGURE 3:**
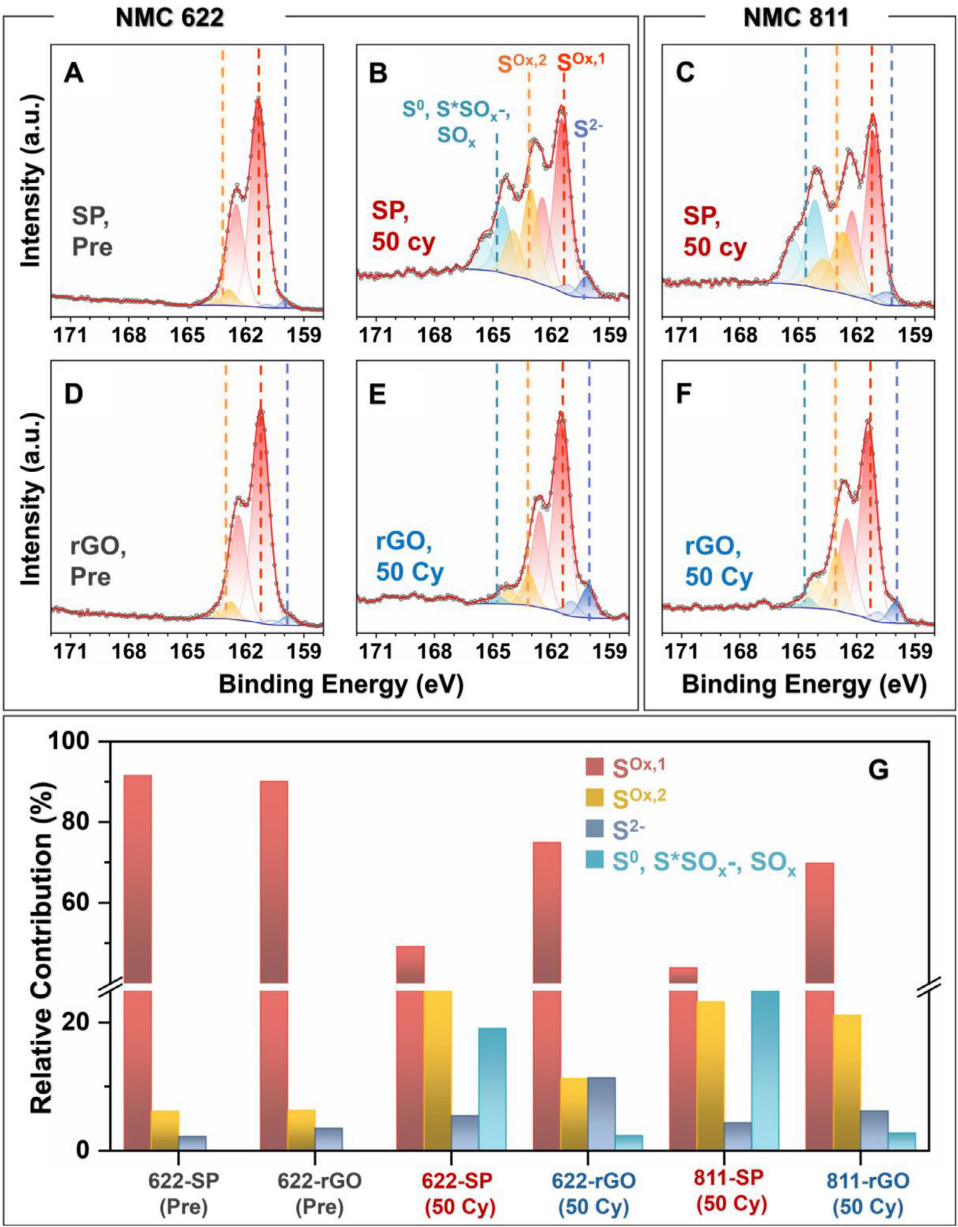
X‐ray photoelectron spectroscopy (XPS) investigation of the cathode interfacial evolution. S 2*p* region XPS spectra for (A,B) NMC622 cathode before cycling (or pre cycling), nearly identical for both (A) SP and (B) rGO‐based cathode, and for NMC622 cathode with (C) SP and (D) rGO as the conductive carbon after 50 cycles. S 2*p* region XPS spectra for NMC811 cathode with (E) SP and (F) rGO after 50 cycles. (G) The relative contribution of the S 2*p* components, corresponding to different sulfur‐containing species in the CEI, inferred from the XPS data in (A‐F). In the (A‐F) fitted XPS data, grey hollow circles represent experimental data, the red solid line denotes the overall fitted data, and the shaded regions stand for fitted individual components as indicated in (B).

**FIGURE 4:**
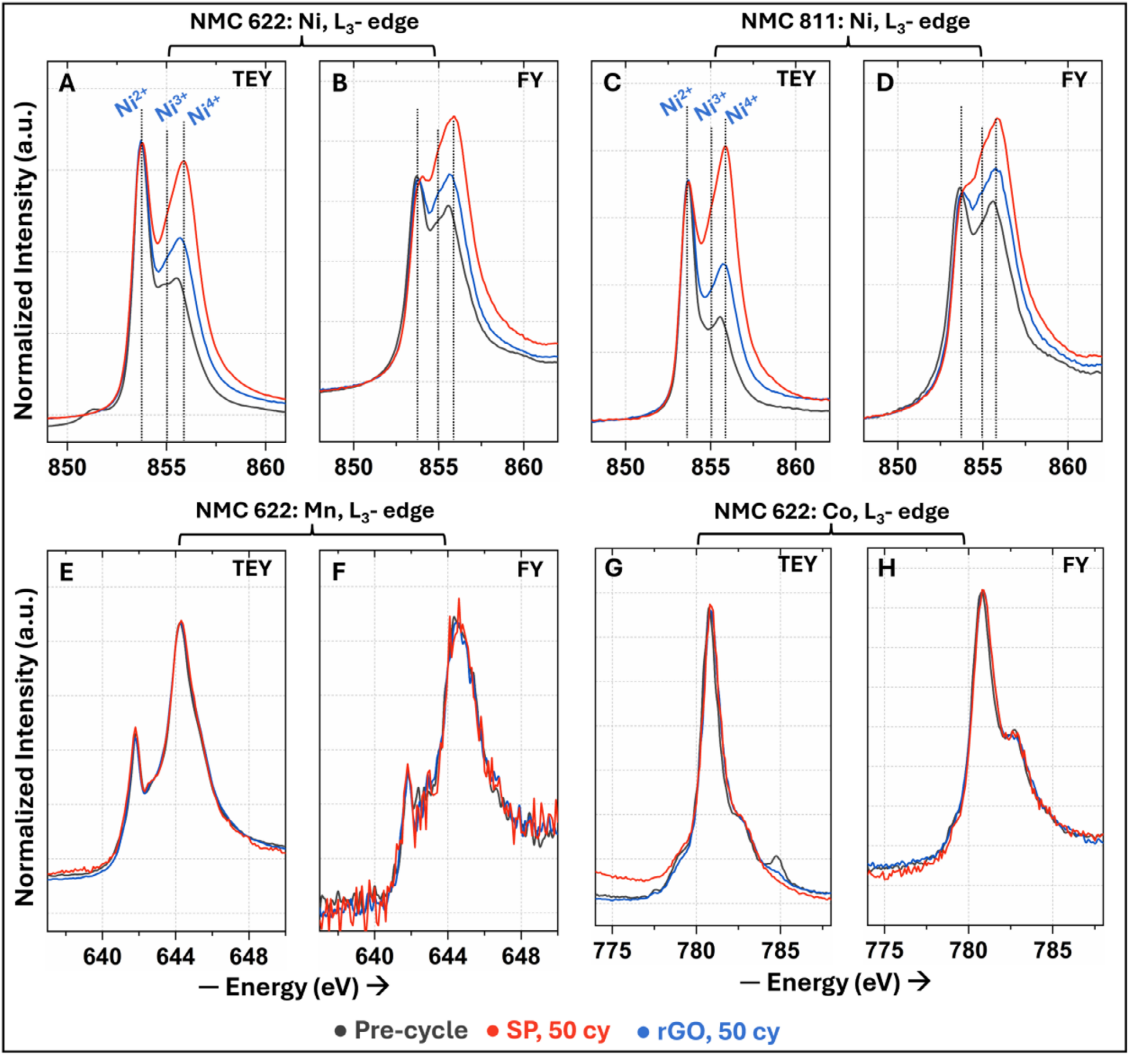
NEXAFS reveals the build‐up of NMC electrochemical inhomogeneity. NEXAFS Ni L_3_‐edge spectra acquired in (A) TEY and (B) FY mode for the SP and rGO‐based NMC622 cathode composites before and after 50 cycles. Ni L_3_‐edge spectra, acquired in (C) TEY and (D) FY mode for the SP and rGO‐based NMC811 cathode before and after 50 cycles. Mn and Co L_3_‐edge spectra in (E,G) TEY and (F,H) FY mode for the NMC622 composite cathode with SP and rGO before and after cycling.

To verify our hypothesis around the role of the oxygen‐functionalized conductive carbon in arresting the Ni‐rich NMC‐based ASSB cell degradation, pristine and cycled cathode composites for both SP and rGO were probed by XPS (Figure 3) and near‐edge X‐ray absorption fine structure (NEXAFS) spectroscopy (Figure 4). Cathode composites were probed after 50 cycles, as by then the dQ/dV signature of the secondary phase becomes prominent. As evident from Figure 3A,B, and Figure S12, there are very minor changes in the S 2*p* (and P 2*p*, Figure S13a–f) region from pristine Li_6_PS_5_Cl to SP and rGO cathode composite prior to cycling—with the most pronounced S 2*p* peak signal (∼162 eV) being that of the PS_4_^3−^ species, labeled as S^Ox,1^ in the spectra [13, 14, 16]. As shown in Figure 3A–D for NMC622 and Figure 3E,F for NMC811, cycling brings out differences between the SP and rGO‐based cathode evolution. Upon cycling, two higher binding energy S 2*p* signatures appear. The one labeled as S^Ox,2^ (S 2*p*3/2: 162–164 eV) can be assigned to polysulfide species such as P_2_S_n_^y−^ and Li_2_S_n_, resulting from Li_6_PS_5_Cl oxidative degradation [41]. There is a notably higher intensity of this component, together with diminished S^Ox,1^ for the SP cell compared to that for rGO. Although previous works by Janek et al., attributed this S^Ox,2^ signal enhancement to primarily an increase in polysulfide species [13, 14], it is essential to note that nickel‐based transition metal sulfides also contribute to the S 2*p* signal in the same region [41, 42]. These sulfides can form upon the degradation of the NMC surface by reactive polysulfides. Such a reaction is also underscored by the pronounced increase in S^*^SO_x_ type species for the SP cathode upon cycling. The broadness of the signal here implies the possibility of overlapping contributions from thiosulfate, sulfate, elemental sulfur, and even metal sulfides [16].

As for NMC622, NMC811 also shows a much higher increase in S^Ox,2^, and S^*^SO_x_ intensity upon cycling with SP carbon compared to that with rGO. A lower binding energy signature (∼160 eV) also appears upon cycling for both SP and rGO, which can be assigned to S^2−^ species in Li_2_S. As shown in our previous work, this forms from the reaction of Li_6_PS_5_Cl with the oxygen functional groups of rGO or the reaction of reactive polysulfides with the NMC surface (SP‐based cathode), both pathways leading to the formation of sulfate and thiosulfate type byproducts. For better comparison, the relative contribution of different S 2*p* components corresponding to different sulfur‐containing species in the CEI is shown in Figure 3G. The P 2*p* region of the XPS mirrors the observation in the S 2*p* region (Figure S13g), highlighting the deterioration of the PS_4_^3−^ component intensity along with a large increase in the intensity of the polysulfide (P_2_S_n_^y−^) and phosphate (PO_x_^y−^) type species in the CEI for both (NMC622 and NMC811) SP‐based cathodes upon cycling.

### 2.4 NEXAFS Suggests Build‐up of NMC Electrochemical Inhomogeneity

NEXAFS at the Ni L_3_‐edge (along with Mn and Co L_3_‐edge and O K‐edge) before and after cycling (100% DOD, 50 cycles) sheds further light on the CEI evolution, specifically highlighting changes to the active NMC material. Transition metal L‐edge spectra directly probe the unoccupied 3d states of the metal's hybridized octahedral crystal field and allow probing of different sampling depths by changing the detection modes [43]. NEXAFS data were collected using Total Electron Yield (TEY), Fluorescence Yield (FY), Partial Electron Yield (PEY), and Auger Electron Yield (AEY) modes, each with distinct surface depth sensitivities. AEY and PEY modes are highly surface‐sensitive, probing only the top ∼1–2 and 2–3 nm of the sample, respectively. TEY mode extends the probing depth up to approximately 5–10 nm, overlapping surface to subsurface level, while FY mode has the deepest penetration, reaching up to 200 nm, providing insights into subsurface states [23, 44]. Regardless of the mode, the Ni L_3_‐edge for pre‐cycled NMC622 shows no notable difference between SP and rGO‐based composites (Figure S14), which agrees with the XPS observation. The Ni L_3_‐edge spectra can be assigned to two prominent features corresponding to Ni^2+^ at 853.6 eV and Ni^4+^ at 856.1 eV. A less pronounced feature in the middle (854.9 eV) is ascribed to Ni^3+^ [44]. Compared to in FY, in the surface‐sensitive (TEY, PEY, AY) modes, an initially higher concentration of Ni^2+^ states can be seen, aligning well with previous reports [44]. After 50 cycles, the SP‐based NMC622 cathode displays a marked increase in higher oxidation state signatures (Ni^3+^/Ni^4+^) in TEY and FY modes (Figure 4A,B), as well as in AEY and PEY modes (Figure S15a,b). The rGO‐based NMC622 cathode not only shows a smaller increase in higher oxidation states (Ni^3+/4+^ feature), but the concentration of Ni^3+/4+^ is also much less near the surface than for the SP‐based cathode (Figure 4A,B). A similar trend is observed for cycled SP and rGO‐based NMC811 cathode composites (Figure 4C,D), with a more pronounced increase in surface Ni^3^^+^/Ni^4^^+^ states for SP‐NMC811 compared to SP‐NMC622. The enrichment of Ni^3+/4+^ spanning the surface and subsurface layers at 100% DOD suggests a progressive nickel redox irreversibility, which can stem from a build‐up in NMC (electro)chemical inhomogeneity. Importantly, unlike Ni L_3_‐edge, Mn and Co L_3_‐edges do not show any variation in signature in both TEY and FY modes for NMC622 (Figure 4E,F) and NMC811 (Figure S16) for both SP and rGO cells. This is consistent with the fact that Mn is well known to be electrochemically inactive, and studies from liquid electrolyte cells have shown that Co also does not exhibit notable redox activity in Ni‐rich NMCs in the applied cycling potential window [45, 46].

To assess changes in TM–O hybridization and interfacial bonding, we examined the pre‐edge region (<535 eV) of the O K‐edge, which captures O 1s → TM 3d‐O 2p transitions [44, 47]. Based on prior TEY O K‐edge NEXAFS studies on NMC622 [44] and NMC811 [47], three key features can be identified (dotted lines) in Figure S15c,d: transition to Ni^3+^ 3*d*‐O 2*p* (528 eV), Mn^4+^/Co^3+^/Ni^3+^ 3*d*‐O 2*p* (530 eV), and Mn^4+^/Ni^2+^ 3*d*‐O 2*p* (532 eV) hybridized states. Even before cycling, O K‐edge features are weak in FY mode due to oxygen's low fluorescence yield and strong self‐absorption in dense oxides, unlike TM L_3_‐edges, which remain prominent across all modes. Nevertheless, all the transitions decrease in intensity upon cycling, more prominently for the SP‐based cathode in the TEY mode. Even in the FY mode, the SP cathode shows a noticeable drop in intensity for all three features, in contrast to that for the rGO cathode. This attenuation of O K‐edge features after cycling suggests a disruption or masking of TM‐O hybridized states, likely caused by the accumulation of interfacial byproducts or CEI. Taken together with TM L_3_‐edge evolutions, the data point toward a CEI‐induced electrochemical inhomogeneity, plausibly involving electrochemical isolation of NMC particles, which displays only partial redox activity with incomplete reduction of oxidized NMC or Ni^4+^ species.

### 2.5 NMR Evidence of NMC Particle Electrochemical Isolation Due to Interfacial Degradation

**FIGURE 5:**
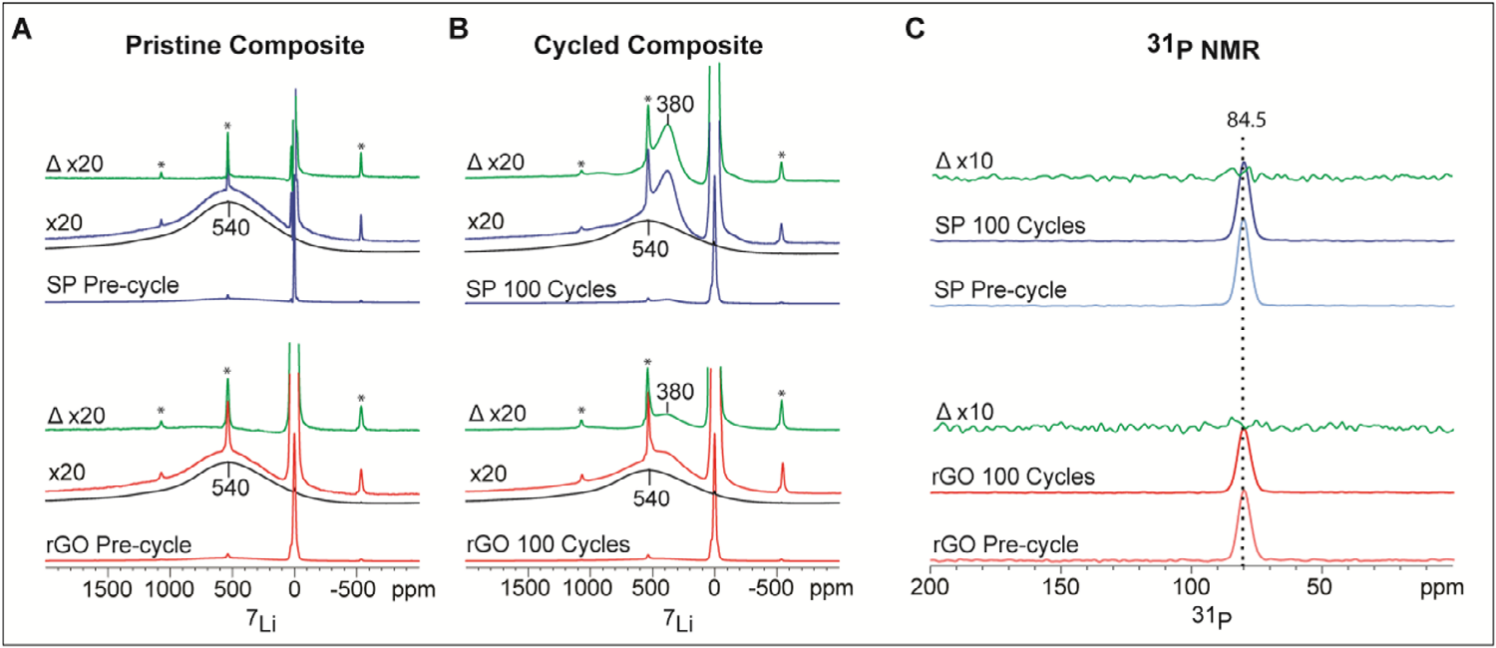
NMR reveals electrochemical trapping of NMC by insulating CEI. (A‐B) ^7^Li NMR spectra of pristine and cycled (100 cycles, 0.2 C, room temperature) NMC622 cathode composites. Red data trace represents rGO‐based NMC622, blue data line corresponds to SP‐based NMC622, black line represents neat NMC622, and green shows the difference between the cathode and neat NMC622 spectra. The “^*^” denotes spinning sidebands. (C) ^31^P NMR spectra of pristine and cycled (100 cycles, 0.2 C, room temperature) NMC622 cathode composites. Light blue and light red data lines correspond to pristine SP and rGO composites, respectively, while blue and red data lines represent the cycled SP and rGO composites. The difference between cycled and pristine spectra is shown in green, with the zoomed‐in region amplified by a factor of 10.

Nuclear Magnetic Resonance (NMR) data further evidence the increase in high oxidation state nickel fraction in the cycled SP‐based cathodes at100% DoD. Figure 5A displays the ^7^Li NMR spectra of the pristine SP and rGO composites, along with neat NMC622. The broadness of the NMC peak at ∼540 ppm arises from the large number of nickel, manganese, and cobalt distribution possibilities around the lithium ions [48, 49], coupled with the paramagnetic relaxation‐induced signal broadening due to the Ni^2+^/Ni^3+^ and Mn^4+^ ions. Depending on the type of transition metal, its oxidation state, and Li─O─TM geometry, the experimentally observed ^7^Li chemical shift is the sum of different hyperfine shifts [49]. Nominally, for Ni only and Mn only environments, the expected ^7^Li NMR shifts would be ∼570 ppm and ∼ 711 ppm (based on LiMn_2_O_3_), respectively. Accordingly, NMC622 would lead to a shift of ∼570 ppm, considering only Ni^2+^ and Mn^4+^ in the coordination shell. The Co^3+^ state is diamagnetic, and its presence reduces the observed ^7^Li NMR shift. The sharp peak at 1.5 ppm in the pristine composite materials corresponds to lithium in the diamagnetic Li_6_PS_5_Cl structure. A scaled difference (green plot line) between the spectrum of the pristine cathode composite and the NMC622 yields only the signal of the Li in the Li_6_PS_5_Cl, indicating no change in the NMC component prior to cycling, irrespective of the carbon additive. This observation is consistent with XPS and NEXAFS data, and confirms that the mixing of NMC, Li_6_PS_5_Cl, and carbon additive without cycling does not lead to any change in the lithium environment of the NMC component of the composite electrodes.

However, for the cathode composites cycled 100 times at a 0.2C at room temperature (Figure 5B), the intensity of the ^7^Li peak at ∼540 ppm is slightly decreased for the rGO cathode and has a significantly larger reduction in the SP‐based cathode. Simultaneously, in both cases, a new ^7^Li peak appears at ∼380 ppm, indicating a clear change in the lithium environment. This is most evident in the scaled difference spectra (green trace), where the ∼380 ppm signal has a much greater intensity for the SP cathode than in the rGO cathode, indicating that the presence of standard carbon black SP accelerates the degradation/structural change of the NMC as compared to the rGO. This new signal could be attributed to a new lithium‐containing phase or to a structural change within the NMC. A more reasonable explanation is the formation of a trapped NMC phase, which gets isolated by an ionically and electronically insulating CEI formed around the NMC [50]. This trapped NMC shows only a partial redox activity, and some Ni always remains in the diamagnetic Ni^4+^ state, which would account for the observed reduction in the ^7^Li NMR shift, and potentially the narrowing of the ^7^Li NMR lineshape [49, 51]. This is also explained by the NEXAFS results showing increased Ni^4+^ content.

Notably, the Li_6_PS_5_Cl peak at 1.5 ppm does not show any significant shift between pristine and cycled composites, indicating that lithium in the bulk of the Li_6_PS_5_Cl phase remains mostly unaffected by cycling. Since NMR is a bulk‐sensitive technique, degradation products confined to the interfacial region remain undetected due to their limited quantity [52]. This interpretation is supported by the ^31^P NMR spectra (Figure 5C), which also show no significant differences between pristine and cycled composites.

The combined electrochemical and spectroscopic evidence reveals a distinct degradation mechanism for Ni‐rich NMCs in sulfide‐SE‐based ASSBs. The emergence of a secondary cathodic dQ/dV feature at a lower potential, unique to Ni‐rich NMCs with conductive carbon SP, signals the formation of a redox‐active secondary phase. XPS confirms the buildup of sulfur‐rich CEI species—including polysulfides, thiosulfate, sulfate, and phosphate‐type byproducts—that are ionically and electronically insulating and contribute to impedance rise with cycling. NEXAFS shows a progressive enrichment of Ni^3+/4+^ states across surface and subsurface layers, which is complemented by the ^7^Li NMR data, revealing a new lithium environment consistent with trapped NMC particles retaining Ni^4+^ even after full discharge. This CEI‐induced electrochemical inhomogeneity stems from interfacial degradation and electrochemical isolation of NMC particles, exacerbated by common conductive carbons like SP, which electronically wire the thermodynamically unstable Li_6_PS_5_Cl particles and promote their electrochemical oxidation into reactive polysulfides. In contrast, a conductive carbon with appreciable surface oxygen functionalization (rGO) forms a thin insulating interphase (CnEI) at the carbon‐Li_6_PS_5_Cl interface, suppressing Li_6_PS_5_Cl oxidation and associated CEI degradation. This enables a robust electron percolation network for NMC particles, facilitating effective utilization, high current capability, stable cycling performance, and excellent thermal stability, as further demonstrated below.

### 2.6 Arresting Interfacial Degradation Enables Stable and High‐Power Ni‐Rich NMC Cathode

**FIGURE 6:**
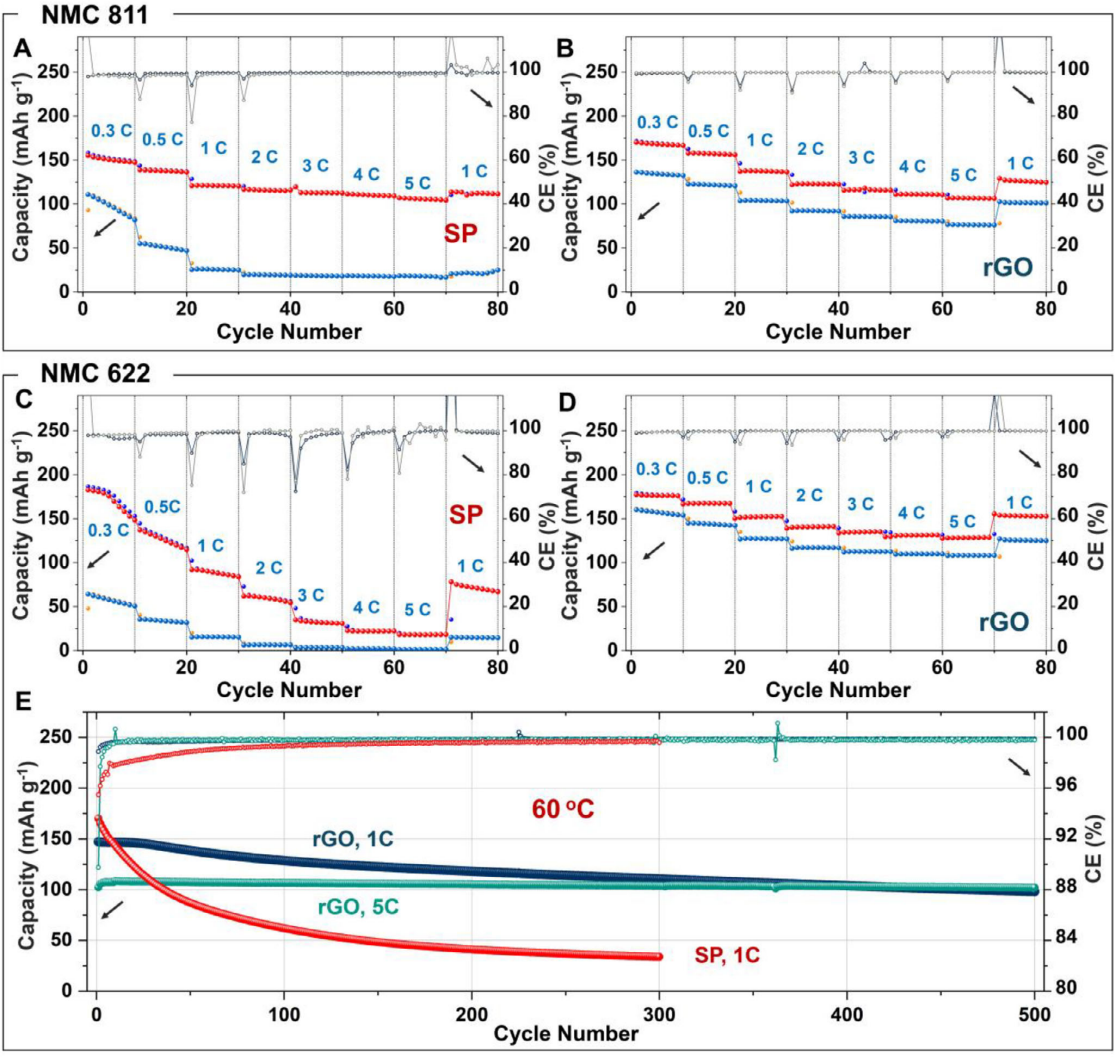
Arresting interfacial degradation enables a stable and high‐power Ni‐rich NMC cathode. Current rate capability for the (A) SP and (B) rGO‐based NMC811 ASSB cells. After cycling at variable current rates, cells were subjected to aging by cycling at a 1C rate for 100 cycles prior to the second round of variable rate cycling. The capacity and CE as a function of cycling for the two rounds of variable rate cycling are shown here. The corresponding data for the NMC622 cell with (C) SP and (D) rGO conductive carbon. (E) Long‐term cycling data for the NMC622 cell with SP and rGO as the conductive carbon. Cells were cycled at a constant temperature of 60 °C. An active NMC loading of ≥12 mg cm^−2^ was applied in all cells unless otherwise specified.

Figure 6A,B, and Table S4, compare the rate capability of NMC811 with the two carbon additives SP and rGO at 60°C across various C rates (0.3C, 0.5 C, 1C, 2C, 3C, 4C, and 5C). High‐temperature cycling not only magnifies the impact of any potential degradations but also allows higher current rate cycling and unveils the extent of (electro)chemical and thermal stability improvement for the engineered system. Here, the cells were cycled 10 cycles at each current rate followed by 100 cycles at 1C before applying the variable rate cycling again with 10 cycles at each current. The intermediate 100 cycles at 1C were performed to probe the effect of aging on the rate capability. It must be noted that while at lower current rates the upper cut‐off (or charge cut‐off) was kept similar to the room temperature study, at higher current rates (2C to 5C), the charge cut‐off was adjusted to account for the polarization effect, as shown in Figures S17–S20. Nevertheless, it is apparent that although the rGO cell displays higher capacities at lower current rates (0.3, 0.5C, 1C), at higher rates (2C, 3C, 4C, 5C), both SP and rGO‐based NMC811 cells show comparable capacities. While this observation can be explained by the significantly higher electronic conductivity of the SP‐NMC811 composite compared to the rGO counterpart, and electronic conductivity being limiting at higher currents, the same explanation does not apparently hold for NMC622 (see below). Anyhow, the rGO cell displays relatively lower decay in capacity at each rate, and much greater retention and current capability during the intermediate 1C cycling (Figures S21 andS22) and subsequent variable rate cycling (Figure 6A,B), respectively. For instance, the SP cell capacity degrades rapidly from 114 to 68 mAh g^−1^ during the intermediate 1C cycling, when the corresponding rGO cell displays 84% (112 mAh g^−1^) retention of the 1st cycle capacity (133 mAh g^−1^). The corresponding dQ/dV evolution of the SP cell, shown in Figure S21b, shows the poorly resolved lower voltage shoulder peak to M → H1 transformation, associated with the limited but polarized redox response from the electrochemically isolated cathode particles. In contrast, the rGO cell maintains an identical dQ/dV profile except for a moderate decay in the dQ/dV peak intensity, a reflection of the small increase in impedance and resulting capacity decrepitation.

Compared to NMC811, NMC622 exhibits a much stronger divergence in rate capability between the SP and rGO cells at 60 °C (Figure 6C,D). While the rGO‐based NMC622 cell shows excellent rate performance with only a modest drop in capacity, delivering 177, 167, 151, 140, 135, 131, and 128 mAh g^−1^ from 0.3C to 5C (Figure 6C,D; Table S4), the SP‐based cell undergoes severe and rapid degradation. Although the SP‐based NMC622 cathode initially delivers a high capacity of 183 mAh g^−1^ at 0.3C, it deteriorates rapidly with increasing current, retaining only 18 mAh g^−1^ at 5C (Figure 6C). Upon reversing the current rate, capacity recovery is limited to 78 mAh g^−1^ at 1C, followed by continued decay during the subsequent 100‐cycle aging period, reaching only 33 mAh g^−1^ at the end of aging. This markedly aggravated cycling‐mediated aging for NMC622, relative to NMC811, is particularly evident during rate testing after the intermediate 1C aging step, where the SP‐based NMC622 cell delivers only 64, 36, and 15 mAh g^−1^ at 0.3C, 0.5C, and 1C, respectively, and nearly zero capacity at higher rates. This behavior highlights pronounced interfacial degradation in the SP‐based NMC622 cathode under elevated temperature cycling, and can be rationalized by its single‐crystalline, smaller particle morphology, which yields a substantially higher NMC–solid electrolyte interfacial contact area compared to the larger, polycrystalline secondary particles of NMC811, thereby accelerating CEI growth and interfacial failure under aggressive conditions.

In addition to morphology‐driven differences in interfacial area, other factors may plausibly contribute to the contrasting high‐temperature rate capability behavior of NMC622 and NMC811 with SP carbon. These include Ni‐content‐dependent oxygen redox and lattice oxygen release at high states of charge [34], which is expected to be more pronounced for NMC811 but is suppressed at room temperature by the lower charge cut‑off employed (Figure 2B; Figure S4). At an elevated temperature (60 °C), however, temperature‐activated surface chemistry can trigger such oxygen redox processes even with a reduced charge potential cut‐off, promoting the formation of a thin insulating phosphate and sulfate‐rich surface layer on Li_6_PS_5_Cl. This passivating layer can arrest redox‑mediated degradation of Li_6_PS_5_Cl and resulting accelerated CEI growth, promoted by conductive carbon additive, thereby helping to explain the comparatively better rate capability and retention observed for NMC811, as also evident from room temperature cycling under an extended charging potential (Figure S4). While these effects are not isolated quantitatively in the present study, they provide a physically consistent framework to rationalize the divergent degradation behavior under elevated‐temperature conditions.

In contrast, besides impressive rate capability, the rGO cell shows resilience against cycling‐mediated aging and degradation, as evident from 140 mAh g^−1^ deliverable capacity after the aging period, a remarkable 91% retention relative to the 1st cycle capacity (in the 100 cycle period) of 154 mAh g^−1^ (Figure S22a and Table S4). The post‐aging rate capability performance highlighted by discharge capacities of 160, 145, 127, 117, 113, 110, and 108 mAh g^−1^ at 0.3C to 5C current rates also reflects the resilience of the rGO‐based NMC622 cell against degradation. As for the NMC 811‐rGO cells, NMC622‐rGO cells, too, do not show any sign of the secondary phase formation in the dQ/dV profile evolution (Figures S22c and S20), which otherwise show up (but poorly resolved) for the SP‐based NMC622 cells during the intermediate 1C rate cycling stage (Figures S22b and S19).

The practical implication of the choice of conductive additive on engineering stable cathode interface is further demonstrated by the excellent long‐term cycling of the NMC 622‐rGO cells at 1C (post five activation cycles at a 0.2C rate at room temperature) and 5C (post the rate capability test presented above) at the elevated temperature of 60^ °C^, which can accelerate aging and degradation (Figure 6E). At 1C, the rGO cell delivers a 1^st^ cycle capacity of 148 mAh g^−1^ with retention figures of 88%, 76%, and 67% of its initial capacity after 100, 300, and 500 cycles, respectively. At the higher rate of 5C, the stability is even better, with a retention of 100%, 97%, and 95% after 100, 300, and 500 cycles, respectively, relative to the initial capacity of 106 mAh g^−1^. Notably, the 5C cell delivers such excellent stability after 250 cycles of rate capability test, which included the intermediate aging of 100 cycles at 1C. In comparison, at the 1C rate, the SP cell capacity dwindles quite rapidly (post five activation cycles at a 0.2C rate at room temperature), showing a retention of only 20% after 300 cycles. The CE data, too, make the stability difference between the SP and the rGO‐based cells glaringly apparent. While the rGO cell shows a high average CE of 99.8% for both 1C and 5C cycling, the SP cell shows a comparatively inferior CE of 99.2%, in agreement with the heightened NMC interfacial degradation, as confirmed by ex situ spectroscopic and in situ impedance analysis.

## 3 Conclusion

This study uncovers a previously unrecognized and far‐reaching implication of the electrochemical degradation of sulfide‐type solid electrolytes on Ni‐rich NMC cathodes. Extensive interfacial degradation of the NMC by sulfide‐SE leads to an electronically and ionically insulating CEI, composed of polysulfides, thiosulfates, and phosphates. Critically, this CEI progressively isolates a considerable fraction of active NMC particles from the electrochemical network, resulting in its incomplete redox and a secondary redox feature in the cathodic branch of the differential capacity (dQ/dV) profile. This unique phenomenon, absent in low‐Ni NMC111 but prominent in NMC622 and NMC811, is confirmed by a combination of in situ impedance‐DRT analysis and ex situ XPS, NEXAFS, and solid‐state ^7^Li NMR of the cathode composites before and after cycling. Replacing conventional carbon black in the cathode formulation with a mildly oxygen‐functionalized conductive carbon (rGO, 2wt% oxygen) arrests both extensive CEI formation and electrochemical isolation. The oxygen‐containing functional groups passivate the carbon‐SE interphase (or, CnEI) that suppresses sulfide SE's redox‐mediated conversion to reactive polysulfides that trigger the NMC degradation. While the improved conductivity of the cathode composite rendered by the conductive carbon boosts active cathode utilization and thus the obtainable capacity, the CnEI‐mediated cathode interfacial stabilization enables enhanced capacity retention and excellent current capability and cycling stability even at an elevated temperature of 60 °C, where interfacial degradation is typically amplified. The improved performance is highlighted by excellent resistance to thermal aging for both NMC622 and NMC811 cathodes, and ∼95% retention of 106 mAh g^−1^ at a 5C rate after 500 cycles for a notably high areal NMC loading of ≥12 mg cm^−2^. Compared to conventional insulating coating of Ni‐rich NMCs, which compromises transport kinetics and thus energy and power capability, functionalized carbon‐mediated in situ regulation of cathode interfacial reactivity presents an easily scalable and performance‐optimized pathway to enable Ni‐rich NMCs in sulfide SE‐based ASSBs. Here, “scalable” refers to a materials‐compatible and process‐agnostic strategy, in which the functionalized conductive carbon is incorporated as conventional carbon black, without introducing additional synthesis or processing steps. Nevertheless, while this work establishes electrochemical isolation as a key degradation pathway, quantifying its absolute bulk fraction and Ni‐content dependence—including the spatial distribution of isolated domains—remains an important next step. Correlative imaging and operando spectroscopies will be valuable to visualize the CEI's spatiotemporal evolution across different cycling regimes.

## 4 Experimental Details

### 4.1 Materials

Li(Ni_0.6_Mn_0.2_Co_0.2_)O_2_ (NMC 622), Li(Ni_0.8_Mn_0.1_Co_0.1_)O_2_ (NMC 811), and Ampcera Li_6_PS_5_Cl were procured from MSE Supplies LLC (AZ, USA). Li_6_PS_5_Cl with a D50 of 80 µm (150 mesh, bulk conductivity of 2–4 mS cm^−1^) was used as the solid electrolyte separator layer, while Li_6_PS_5_Cl with a D50 of 1 µm (bulk conductivity of 1.2–1.5 mS cm^−1^) was utilized for cathode composite preparation. Super P (TIMCAL), known for its high electronic conductivity, served as the reference conductive additive material. Super P (denoted as SP here) was annealed at 1000 °C for 1 h with a heating rate of 5 °C min^−1^ under a 20 mL min^−1^ argon flow in a tube furnace to remove any residual surface oxygen functionalities. As the modified carbon, commercial rGO annealed at 1000 °C under conditions similar to the SP carbon was employed.

### 4.2 Preparation of Cathode and Anode

NMC 622 and NMC 811 were vacuum‐dried at 200 °C for 24 h in a Büchi oven before being utilized as cathode active materials. To ensure phase purity and confirm the morphology of the NMC material, X‐ray diffraction and scanning electron microscopy were performed before and after the thermal pretreatment. Using an agate mortar, the cathode composite was prepared by manually grinding NMC622/NMC811 with Li_6_PS_5_Cl in a 70:30 weight ratio. Subsequently, 100 mg of this blend was ground with 6 mg of the carbon additive, resulting in a final composition of NMC622/NMC811: 66 wt.%, Li_6_PS_5_Cl: 28.3 wt.%, and Conductive additive: 5.7 wt.%. The Li_x_In (0.5<x<1) anode was produced by pressing and rolling Li and In foils. Uniform LixIn coins, 100–150 µm thick and 7 mm in diameter, with an average Li content of 1.5–2 mg, were punched out and utilized as the anode.

### 4.3 Fabrication of the All‐Solid‐State Battery (ASSB) Cells

The ASSBs were assembled in an argon‐filled glovebox with H_2_O levels maintained below 0.1 ppm and O_2_ levels below 1 ppm. A custom‐made cell setup comprised of the cell body with an internal diameter of 12 ± 0.02 mm was employed. The cell is capable of ensuring a controlled assembly and stack pressure. To construct the cell, 90–100 mg of solid electrolyte (SE), specifically Li_6_PS_5_Cl (150 mesh, D50 ∼ 80 µm), was initially pressed uniaxially at about 256 mPa for 1 min to form the SE layer in the die. After this, ≥20 mg of the freshly prepared cathode composite was added to the SE layer and pressed at an uniaxial pressure of approximately 512 mPa for 1 min. The Li_x_In coin was then placed on the anode side plunger, i.e., the other side of the SE layer, to complete the electrode stack. The anode and cathode plungers, equipped with O‐rings, ensured airtight sealing during cycling. After the assembly, the cells were transferred out of the glovebox and placed in a custom compression jig under a constant uniaxial stack pressure of 45–49 mPa and electrochemically cycled [53, 54].

### 4.4 Galvanostatic Cycling

Experiments were conducted at both room temperature (20 ± 2 °C) and an elevated temperature of 60 °C using a VMP3 potentiostat/galvanostat (BioLogic). Some galvanostatic cycling data at room temperature were also obtained using the LAND CT3002A battery cycler. The galvanostatic cycling protocol involved charging to a voltage limit of 3.7 V for NMC 622 and 3.6 V for NMC 811, relative to Li_x_In (where 0.5 ≤ x ≤ 1.0) and discharging to a cut‐off of 2 V relative to Li_x_In. Electrochemical impedance spectroscopy (EIS) data were recorded over a frequency range of 1 mHz to 10 µHz with a 20 mV signal amplitude. The resulting EIS spectra were analyzed and fitted using the ‘distribution of relaxation time’ (DRT) protocol. For EIS measurements taken during cycling at 100% state‐of‐charge (SOC) and 100% depth‐of‐discharge (DOD), cells were allowed to rest for 2 h before the AC perturbation was applied.

Cumulative inefficiency has been defined as a parameter to provide a cumulative measure of the Coulomb loss, which offers a comprehensive assessment of degradation over extended cycling [15]. It is calculated based on the following equations:

(1) $$\mathrm{Inefficiency}\;=\frac{100\;-\;\mathrm{Coulombic}\;\mathrm{efficiency}\;\left(\%\right)}{100}$$

(2) Cumulativeinefficiency=∑Inefficiency

These equations define the inefficiency on a per‐cycle basis and aggregate it over multiple cycles to yield the cumulative inefficiency. This approach captures subtle changes in Coulombic efficiency that may not be apparent when considering individual cycles alone.

Additional experimental details regarding DRT and spectroscopy analyses are provided in the Supporting Information.

## Conflicts of Interest

A patent application (PCT) has been filed that encompasses some of the innovations discussed in this study.

## Supporting information




**Supporting File**: advs74092‐sup‐0001‐SuppMat.pdf.

## Data Availability

The data that support the findings of this study are available from the corresponding author upon reasonable request.;
