# Supplementary material for: Mitigating Electrochemical Isolation in Ni‐Rich Layered Cathodes for Durable Solid‐State Batteries
Source: Adv Sci (Weinh). 2026 Jan 28;13(19):e18327. doi: 10.1002/advs.202518327 (PMC13045493; doi:10.1002/advs.202518327)
Supplement: Supplementary file 1 — Supporting File: advs74092‐sup‐0001‐SuppMat.pdf. [file ADVS-13-e18327-s001.pdf]

Mitigating Electrochemical Isolation in Ni-Rich Layered Cathodes for Durable Solid-State Batteries

*Abhirup Bhadra[a], Maxime Brunischolz[a], Aditya Rawal[b], Jacob Otabil Bonsu[a], Tongjun Luo[c], Lars Thomsen,[d] Wesley M. Dose[c], Dipan Kundu[a,]**

[a] School of Chemical Engineering, UNSW Sydney, Kensington, NSW 2052, Australia

[b] *MWAC, UNSW Sydney, Kensington, NSW 2052, Australia*

[c] School of Chemistry, The University of Sydney, Sydney, NSW 2006, Australia

[d] Australian Synchrotron, ANSTO, Clayton, VIC, 3168 Australia

*Corresponding author; Email: d.kundu@unsw.edu.au

Additional Experimental Details:

Distribution of Relaxation Time (DRT) Analysis. The Python-based pyDRTtools software, developed by Francesco Ciucci's lab (University of Bayreuth), was used to deconvolute the EIS data, utilizing second-order Gaussian radial basis functions (RBF) with a shape factor control ranging from 0.5 and a regularization parameter set at 0.001. The RBFs employed by pyDRTtools are defined as $\phi\mu(x) = \exp(-(\mu x)^2)$, where $x = |\ln \tau - \ln \tau m|$, and μ represents the shape factor.[1-3] The segment assignments have been conducted based on previous work by Orue Mendizabal et al, Bhadra et al and Bonsu et al.[4-6]

X-ray photoelectron spectroscopy (XPS) measurements were conducted using a Thermo Scientific™ ESCALAB™ 250Xi XPS Microprobe instrument, employing monochromatic Al Kα radiation (1486.6 eV) at 120 W power as the excitation source. The beam voltage was set at 13.8 kV, with a spot size of 500 μm, and the chamber vacuum was maintained below 2×10^{-9} bar. Samples were transferred to the XPS chamber under an argon atmosphere, and the sample surfaces were cleaned using Ar^+ sputtering (1 keV) for 1 minute. XP spectra were calibrated against the binding energy standards of Au 4*f*7 = 83.96 eV, Ag 3*d*5 = 368.21 eV, and Cu 2*p*3= 932.62 eV,with adventitious carbon at 284.8 eV serving as the reference. CasaXPS software was used for spectral fitting. All the XP spectra were fitted using a Shirley-type background. Gaussian-Lorentzian (GL) or Asymmetric Lorentzian (LA) line shapes were used as fitting functions. The S 2*p* spectra were fitted with $2p_{3/2}$ and $2p_{1/2}$ doublets under three constraints: (a) a 1.18 eV separation between peaks, (b) a 2:1 area ratio for $2p_{3/2}$ to $2p_{1/2}$, and (c) identical FWHM values. P 2*p* doublets were fitted similarly, with a peak separation of 0.85 eV between $2p_{3/2}$ and $2p_{1/2}$.

Soft X-ray Absorption Spectroscopy (SXAS) / Near-Edge X-ray Absorption at Fine Structure (NEXAFS). The NEXAFS measurements of the samples were performed using soft X-ray absorption spectroscopy (sXAS) at the soft X-ray beamline of the Australian Synchrotron.[7] These measurements focused on the Ni, Co, and Mn L-edges, as well as the O K-edge. Spectra were acquired using Auger electron yield (AEY), partial electron yield (PEY), total electron yield (TEY), and fluorescence yield (FY) modes, with an energy step size of 0.1 eV.[8]

The probing depths for these modes differ significantly, with AEY probing within ~1–2 nm, PEY around ~5 nm, TEY within ~5–10 nm, and FY up to ~200 nm. This enables a comprehensive

understanding of both surface and bulk characteristics of the Ni-rich NMC-based cathodes. AEY and PEY provide highly surface-sensitive information, while TEY offers near-surface details. FY, being bulk-sensitive, provides deeper insights, although self-absorption can cause distortions and reduce the signal-to-noise ratio.

All NEXAFS spectra were normalized and processed using the **QANT software** made available by the Australian Synchrotron (ANSTO).[9] The data were normalized to the incident X-ray flux (I_0) and peak maxima for accurate comparisons. The I_0 signal, measured using a gold mesh with ~90% transmission, ensures flux-independent results by correcting for variations in beam intensity.[8]

To minimize the effects of surface oxidation caused by moisture or atmospheric oxygen, the Ni-rich NMC-based cathode samples were stored and transported in argon-filled bags, reducing air exposure and preserving their pristine states.

Solid-state 7Li NMR. The solid-state NMR spectra were acquired on a Bruker Neo 300 with a 7 Tesla widebore magnet, operating at frequencies of 116.7 MHz and 121.5 MHz for the ^{7}Li and ^{31}P nuclei. For the measurements, approximately 3 mg of sample was packed in the glove box under an inert argon environment, into 1.3 mm MAS zirconia rotors fitted with vespel in caps and spun to 62.5 kHz and 30kHz for the ^{7}Li and ^{31}P, respectively, at the magic angle in a Bruker double resonance HX widebore probe head, under dry N_2 gas. The ^{7}Li spectra were acquired with a solid echo sequence with pulse lengths of 2.25µs, 5ms recycle delay, and 128k signal transients co-added for sufficient signal-to-noise. The ^{31}P spectra were acquired with a spin-echo sequence with a 2.49µs 90° pulse length, 10s recycle delay, and 512 signal transients co-added for sufficient signal-to-noise. The ^{7}Li chemical shifts were referenced to aqueous LiCl using solid LiCl as a secondary reference, while the ^{31}P chemical shifts were referenced to aqueous H_3PO_4 using ammonium dihydrogen phosphate as a secondary reference.

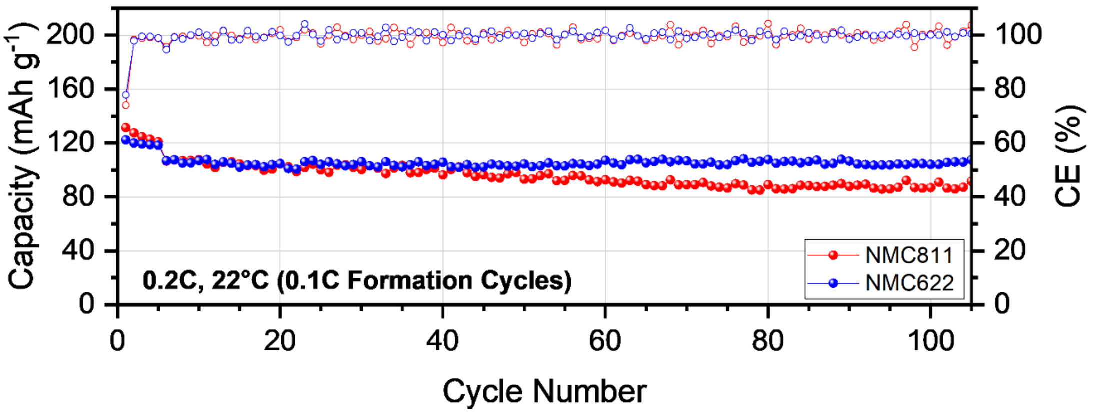

Figure S1. Galvanostatic cyclability data for the NMC622 (blue) and NMC811 (red) -based ASSB cells without any conductive additive in the cathode. After the initial formation cycles at a 0.1 C rate, the cycling was performed at a 0.2 C constant current, where 1 C = 150 mAh g^{-1}, within a 2.0-3.7 V (w.r.t. $Li_{0.5}In$) window at 20±2°C with an active NMC loading of ~12 mg cm^{-2}. $Li_{0.5}In$ served as the anode. The lower specific capacity observed here compared to that shown in Figure 1 and 2 can be rationalized by the absence of conductive carbon in the cathode, which leads to inferior active cathode (NMC) utilization.

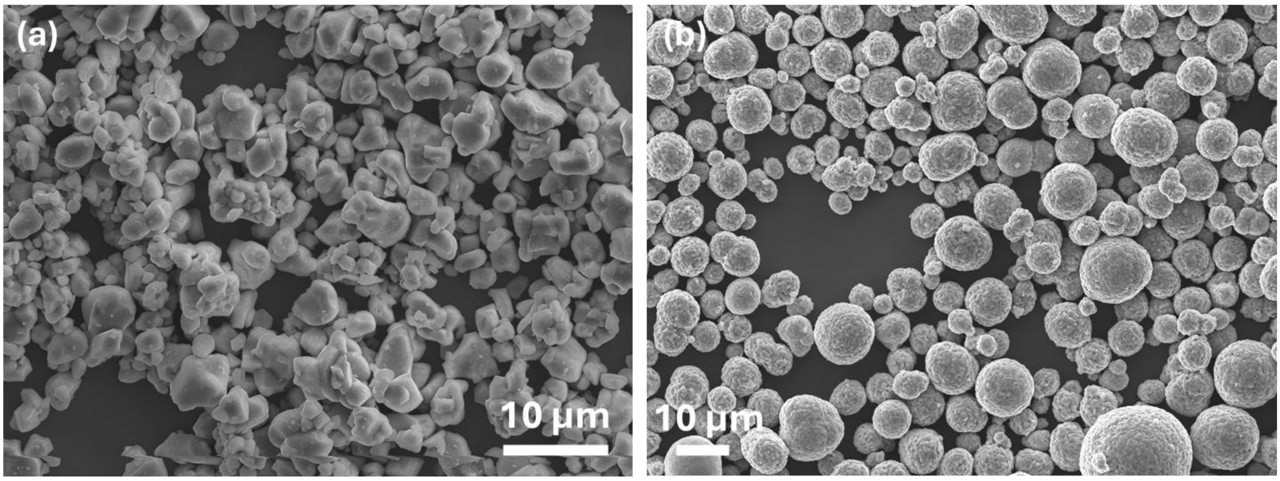

Figure S2. Representative SEM image of the (a) NMC622 and (b) NMC811 material used in this study. Evidently, NMC622 is composed of single-crystalline particles with an average size of 2–5 µm, while NMC811 consists of polycrystalline secondary particles (~5–13 µm) formed by the agglomeration of smaller primary crystallites.

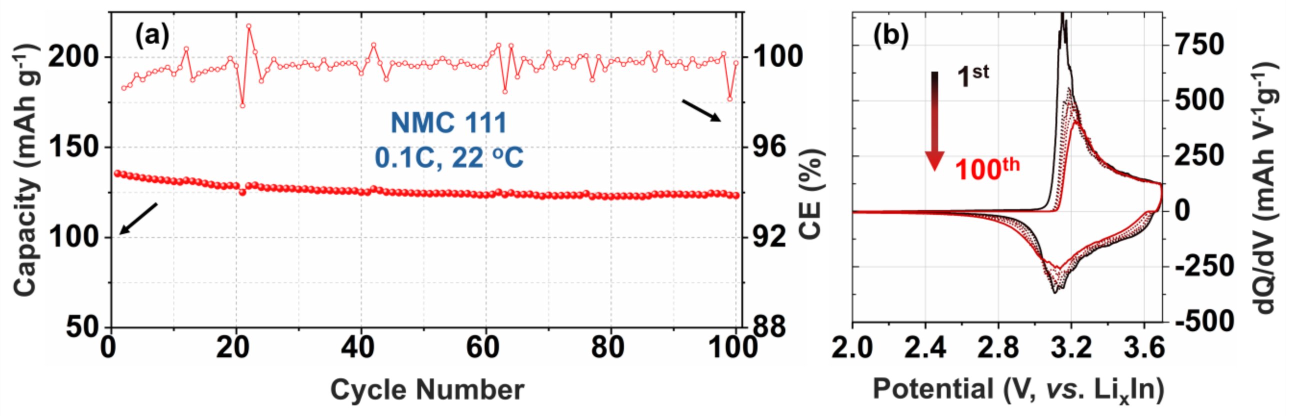

Figure S3. (a) Galvanostatic cyclability data for the NMC111-based ASSB cell with super P or SP carbon as the conductive additive in the cathode and (b) evolution of the corresponding dQ/dV profile as a function of cycling for 100 cycles. The cycling was performed at a 0.2 C constant current, where 1C = 150 mAh g^{-1}, within 2.0-3.7 V (w.r.t. $Li_{0.5}In$) window at 20±2°C with an active NMC loading of ~12 mg cm^{-2}.

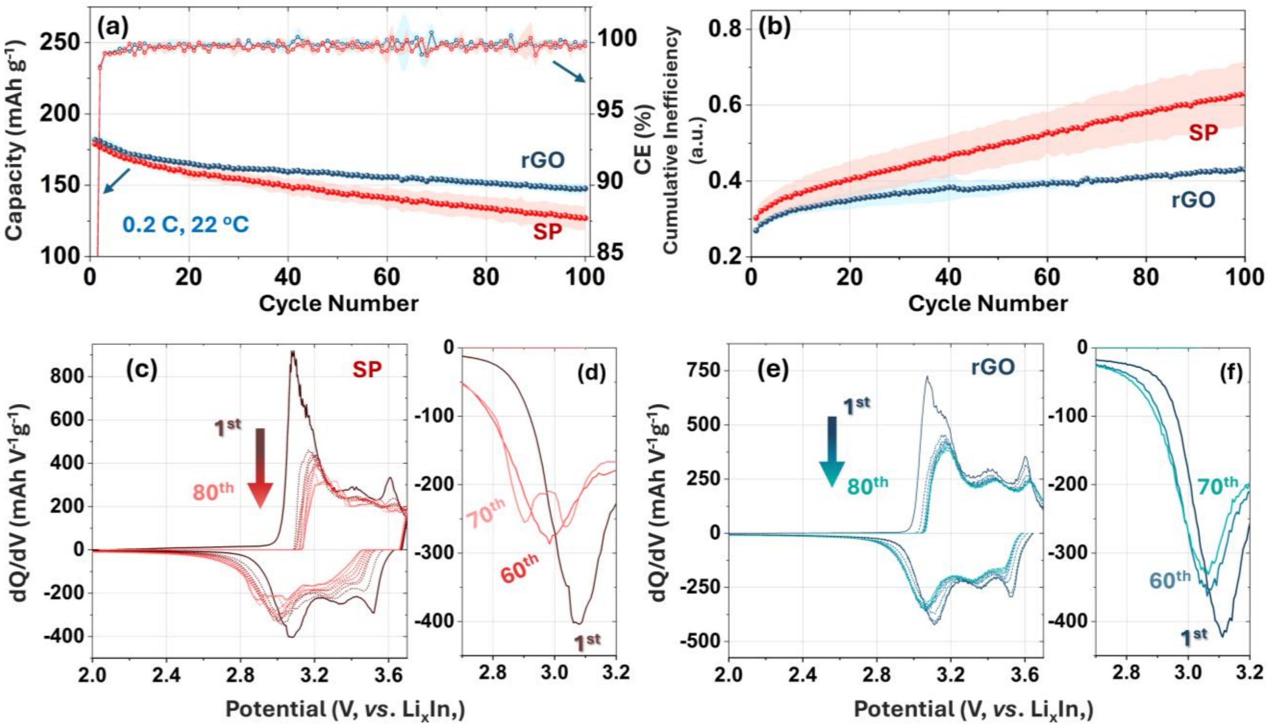

Figure S4. (a) Galvanostatic cyclability of the NMC811-based ASSB cells with Li_6PS_5Cl SE and (b) the corresponding cumulative inefficiency evolution as a function of cycling for the SP carbon-based composite cathode vis-à-vis the rGO-based cathode. The cycling was performed at a constant current of 0.2 C-rate, where 1C = 170 mAh g^{-1}, within 2.0-3.7 V (w.r.t. $Li_{0.5}In$) window at 20±2°C with an active NMC loading of ~12 mg cm^{-2}. The corresponding dQ/dV evolution for the cells with (c, d) SP and (e, f) rGO as the conductive additive in the cathode composite. The color coding for the different carbon additive-based samples is as follows: rGO (Navy Blue) and SP (Red).

Interestingly, with a 3.7 V (against $Li_{0.5}In$) charge voltage cut-off, i.e., for cycling in the 2 – 3.7 V window, which promotes H2→H3 phase transformation, the macroscopic capacity decay for the SP-based cathode is less severe than for cycling in 2 – 3.6 V window, even though the rGO cell still displays greater stability. Furthermore, the onset of the differential capacity signature of the secondary phase formation also gets delayed within this extended potential window. It is believed that the high voltage transformation, along with oxygen redox, modulates the susceptibility towards polysulfide-mediated degradations at the NMC811-Li_6PS_5Cl interface. While an in-depth investigation of this intriguing phenomenon requires a separate thorough probing, the preliminary observation is presented **Figure S4**.

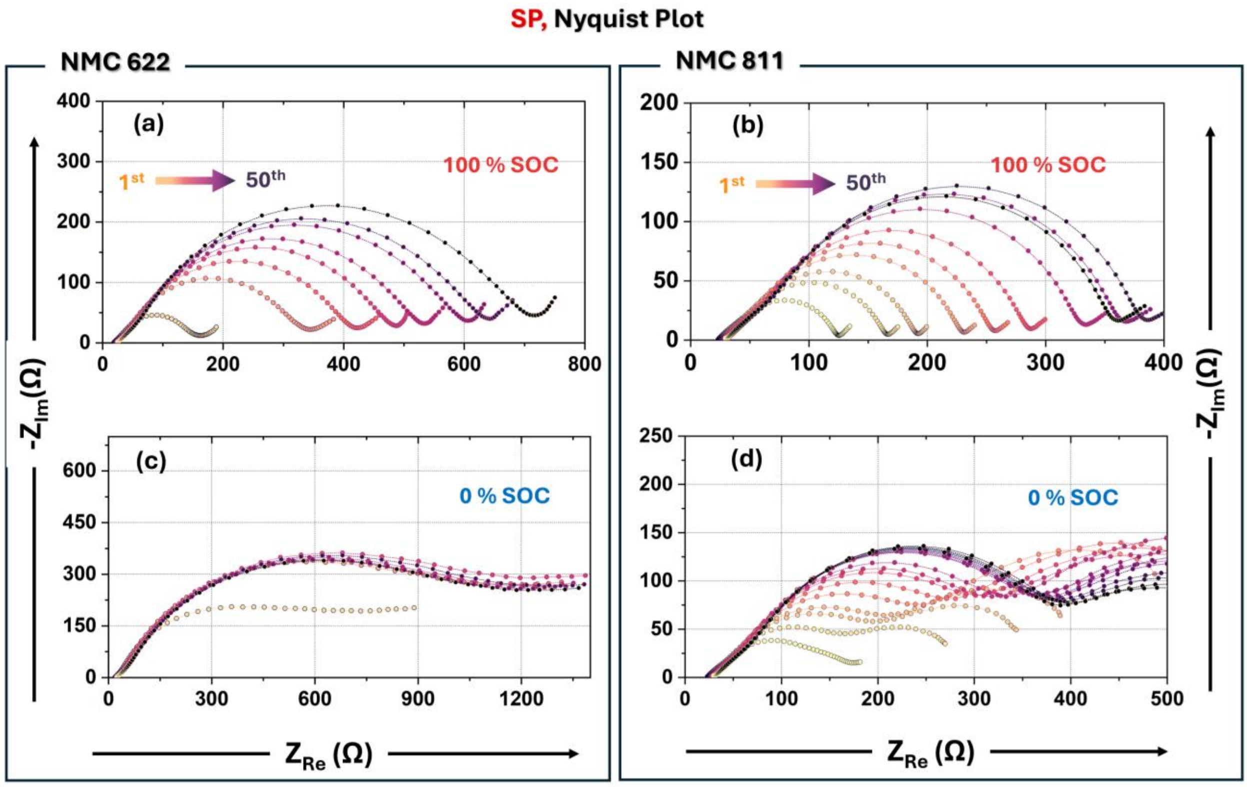

Figure S5. Nyquist impedance evolutions for (a, c) NMC622 and (b, d) NMC811 based ASSB cells with SP as the carbon additive in the cathode, during at 0.2 C constant current cycling (at 20±2°C) over 50 cycles, recorded every 7 (seven) cycles (a, b) in the fully charged or 100% SOC state and (c, d) in the fully discharged or 0% SOC state.

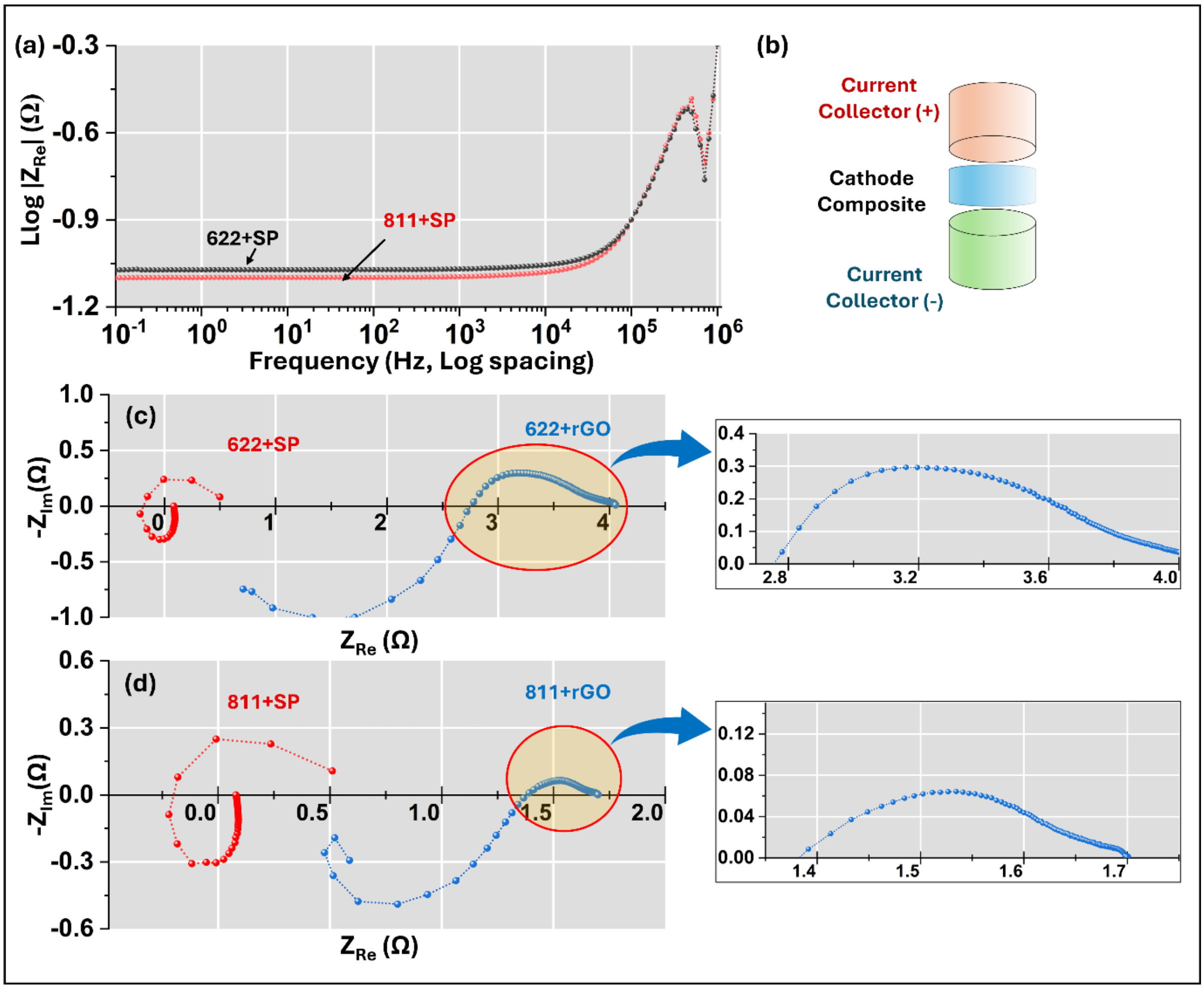

Figure S6. Measurement of conductivity of various cathode composites. (a) The Bode Plot of the composite comprising NMC 811 and NMC 622 with SP and rGO as the conductive additives and Li_6PS_5Cl SE. (b) The blocking electrode configuration used to measure the EIS of the composites. Nyquist Impedance plot for (c) NMC622-rGO vis-à-vis NMC622-SP composite and (d) NMC811-rGO vis-à-vis NMC811-SP composite

Table S1. Conductivity of the cathode composites.

Sample	Z_{Re} (Ohm)	Thickness (cm)	Area (cm^2)	Sigma (S/cm)	mS/cm
**811+ SP**	0.079	0.039	1.13	0.434	434
**811 + rGO**	1.39	0.044	1.13	0.028	28
**622+ SP**	0.085	0.037	1.13	0.387	387
**622 + rGO**	2.78	0.045	1.13	0.014	14

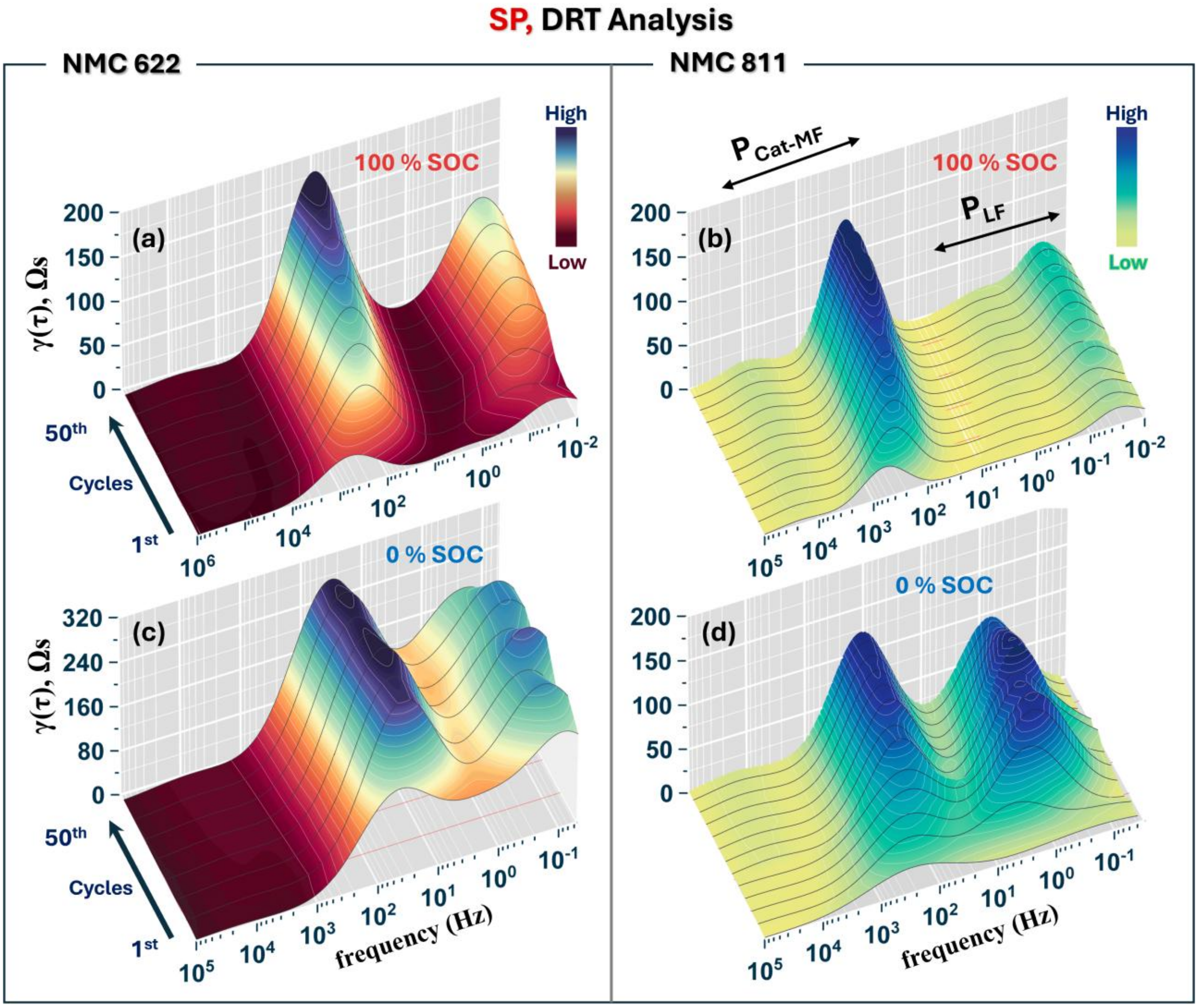

Figure S7. Distribution of relaxation time (DRT) evolutions for (a, c) NMC622 and (c, d) NMC811 based ASSB cells at (a, b) 100% SOC and (c, d) 0% SOC with the SP as the carbon additive during 0.2 C rate cycling (at 20±2°C) over the first 50 cycles recorded every 7th cycle.

Table S2. Measurement of electronic conductivity of carbon additives.

**Sample**	**Z_{Re} (Ohm)**	**Thickness (cm)**	**Area (cm^2)**	**Sigma (S/cm)**	**mS/cm**
**rGO (annealed at 1000 °C)**	0.019	0.046	1.13	2.161	2161
**SP**	0.038	0.045	1.13	1.036	1036

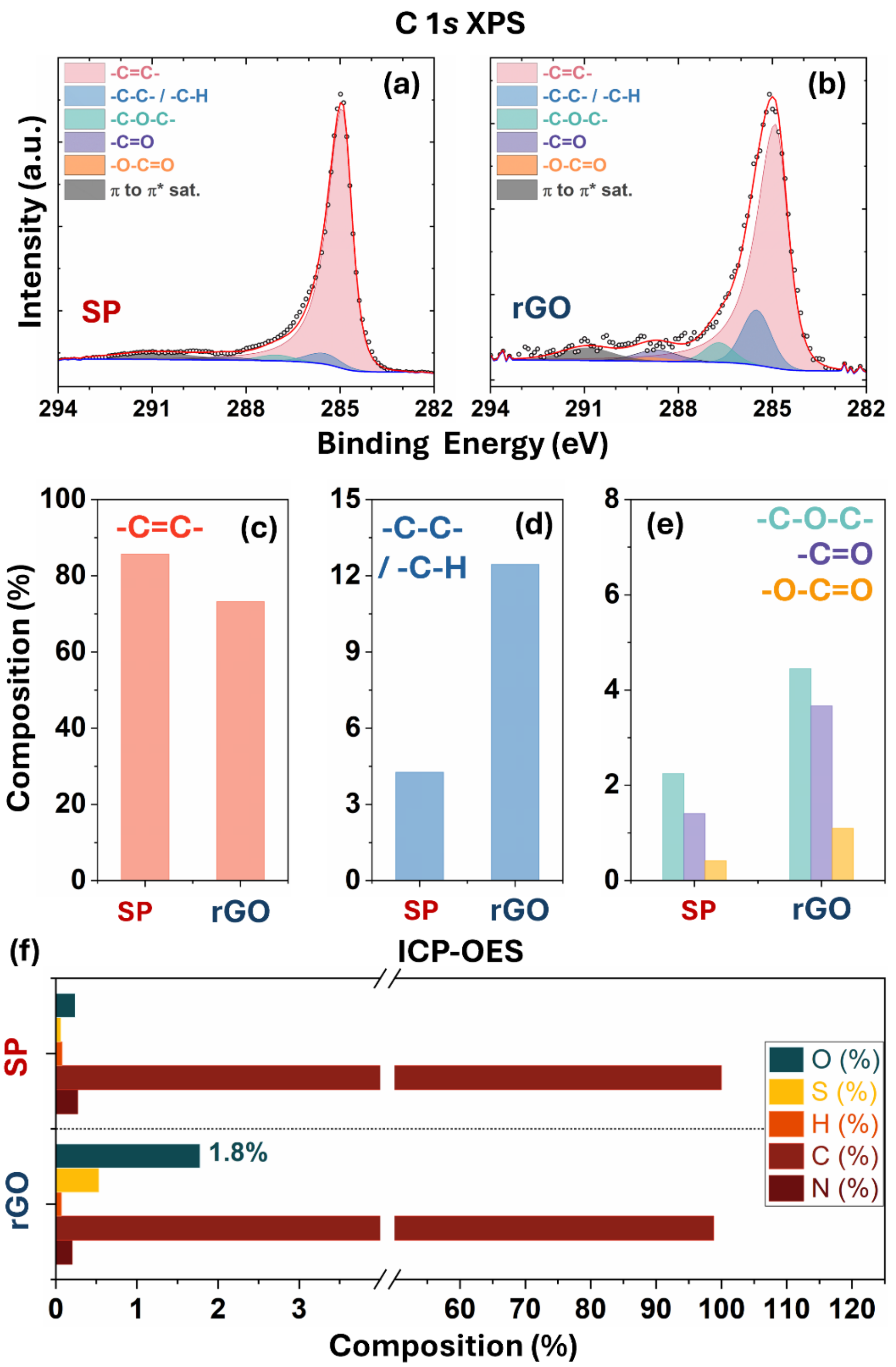

Figure S8. Carbon 1*s* XPS of conductive additives (a) SP, and (b) rGO (annealed at 1000 °C). In the fitted XPS spectra, black data points: experimental data, red line: overall fitted data, and other shaded regions: fitted individual components as indicated on the top-left corner of each plot. The relative contributions of different C 1*s* XPS components corresponding to (c) C=C, (d) C-C/C-H, and (e) COC/CO/OCO groups for SP and rGO. (f) Relative weight% of O/S/H/C/N in SP and rGO obtained from C-H-N-S-O composition analysis of the carbon additives.

Table S3. Relevant parameters corresponding to the room temperature cycling data

Cell Type		C-rate	1st Cycle Discharge capacity (mAh g^{-1})	1st Cycle C.E. (%)	Average C.E. (without 1st cycle) (%)	Retention at 100th Cycle (%)
622-SP	Cell 1	0.2	148.2	68.4 %	99.2 %; (99.6 %)	66 %
	Cell 2	0.2	143.6	69.8 %	99.2 %; (99.5 %)	63 %
	Cell 3	0.2	149.2	70.5 %	98.8 %; (99.1 %)	43 %
	**Cell Avg.**	**0.2**	**147**	**69.6 %**	**99.1 %; (99.4 %)**	**57 %**
622-rGO	Cell 1	0.2	146.2	77 %	99.6 %; (99.8 %)	89 %
	Cell 2	0.2	154.3	75.7 %	99.5 %; (99.8 %)	91 %
	Cell 3	0.2	150.2	75.3 %	99.5 %; (99.7 %)	82 %
	**Cell Avg.**	**0.2**	**150.2**	**76 %**	**99.5 %; (99.8 %)**	**87 %**
811-SP (UCV 3.6V w.r.t. Li0.5In)	Cell 1	0.2	152.7	60.9 %	98.8 %; (99.2 %)	41 %
	Cell 2	0.2	157.2	62.6 %	98.8 %; (99.2 %)	47 %
	**Cell Avg.**	**0.2**	**155**	**61.8 %**	**98.8 %; (99.2 %)**	**44 %**
811-rGO (UCV 3.6V w.r.t. $Li_{0.5}In$)	Cell 1	0.2	154.9	69.7 %	99.5 %; (99.8 %)	76 %
	Cell 2	0.2	155.8	73.5 %	99.5 %; (99.8 %)	73 %
	**Cell Avg.**	**0.2**	**155.3**	**71.6 %**	**99.5 %; (99.8 %)**	**75 %**
811-SP (UCV 3.7V w.r.t. Li0.5In)	Cell 1	0.2	178.6	67.6 %	99.3 %; (99.6 %)	68 %
	Cell 2	0.2	182.4	69.1 %	99.3 %; (99.6 %)	74 %
	Cell 3	0.2	175.9	72.4 %	99.4 %; (99.7 %)	70 %
	**Cell Avg.**	**0.2**	**179**	**69.7 %**	**99.3 %; (99.6 %)**	**71 %**
811-rGO (UCV 3.7V w.r.t. $Li_{0.5}In$)	Cell 1	0.2	177.8	74 %	99.6 %; (99.8 %)	83 %
	Cell 2	0.2	182.5	73.7 %	99.6 %; (99.8 %)	83 %
	Cell 3	0.2	185.1	71.5 %	99.6%; (99.9%)	79 %
	**Cell Avg.**	**0.2**	**181.8**	**73.1 %**	**99.6 %; (99.8 %)**	**81 %**

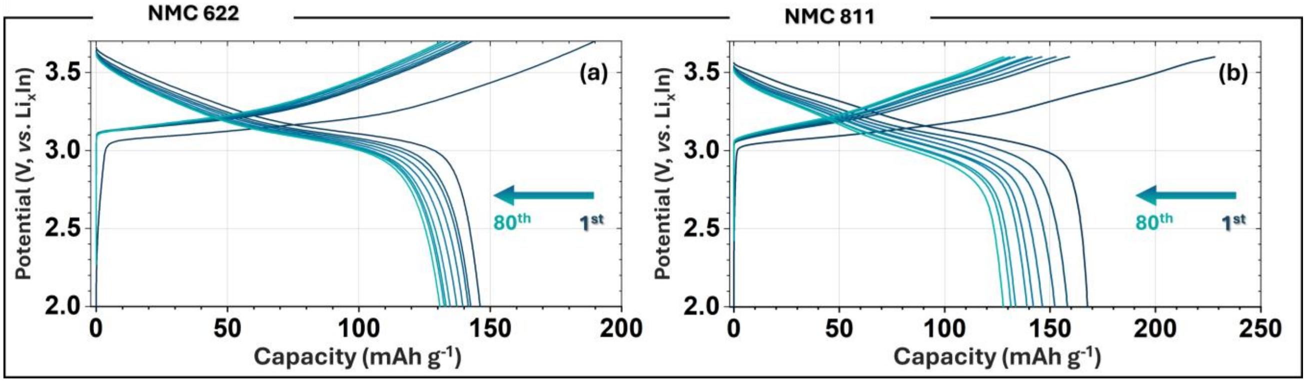

Figure S9. Evolution of the galvanostatic polarization profile for (a) NMC622 and (b) NMC811-based ASSB cell with rGO carbon cathode additive. The cell was cycled at a 0.2C (1C: 160 mAh g^{-1}) at room temperature (20±2°C).

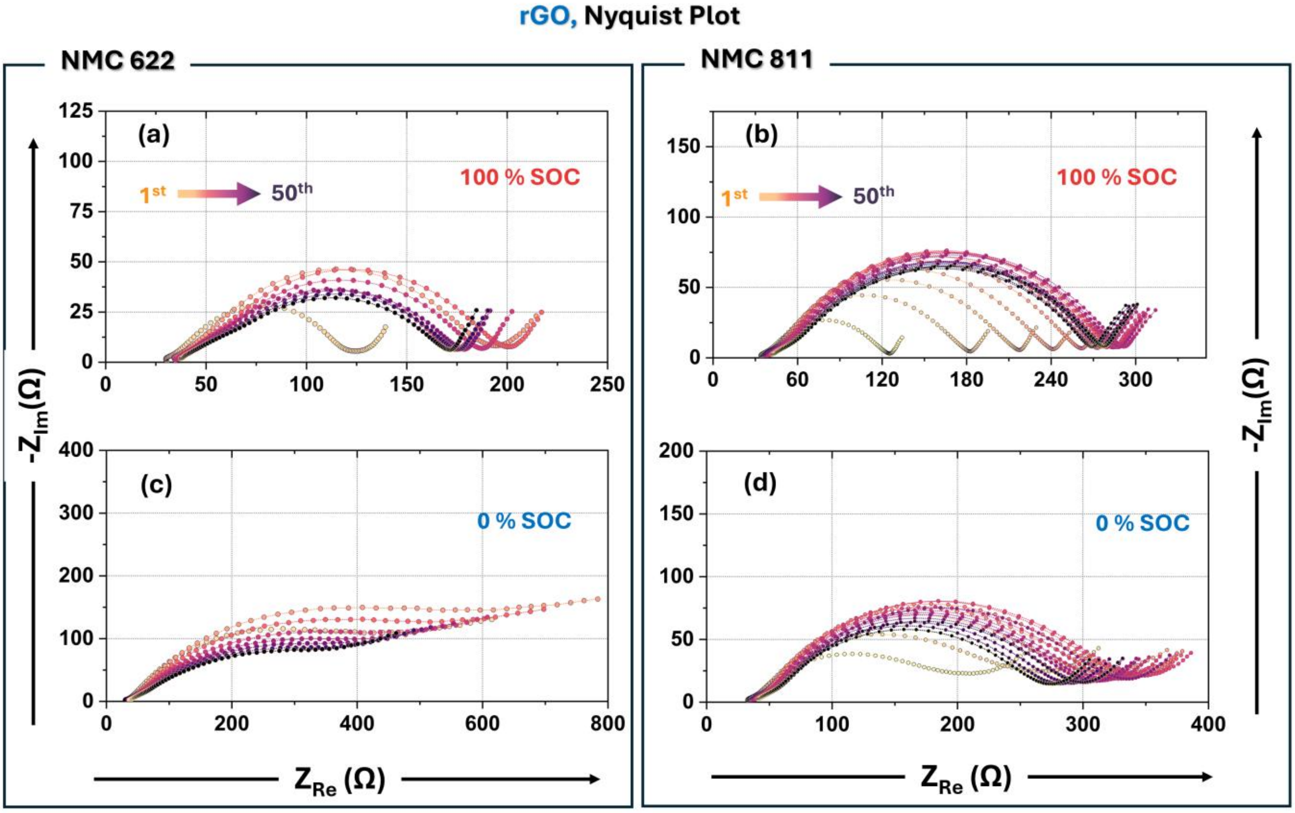

Figure S10. Nyquist impedance evolutions of (a, c) NMC622 and (b, d) NMC811 ASSB cells with rGO as the conductive cathode additive during 0.2 C constant current cycling (at 20±2°C), recorded every 7 cycles in (a, b) the charged or 100% SOC and (c, d) 100% DOD or 0% SOC.

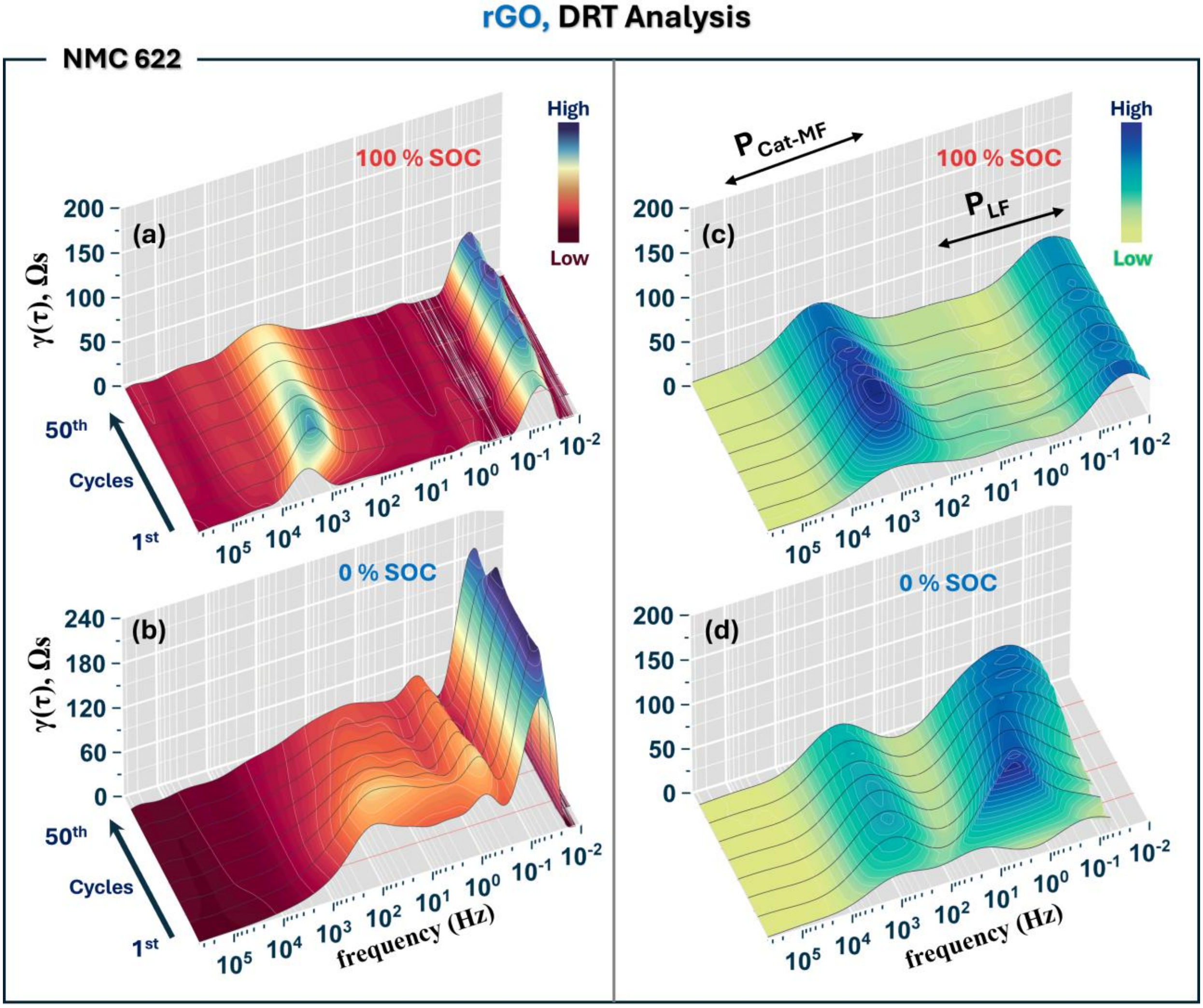

Figure S11. Distribution of relaxation time (DRT) evolutions for (a, b) NMC622 and (c, d) NMC811 based ASSB cells at (a, c) 100% SOC and (c, d) 0% SOC with the rGO as the conductive cathode additive during 0.2 C rate cycling (at 20±2°C) over the first 50 cycles, recorded every 7th cycle.

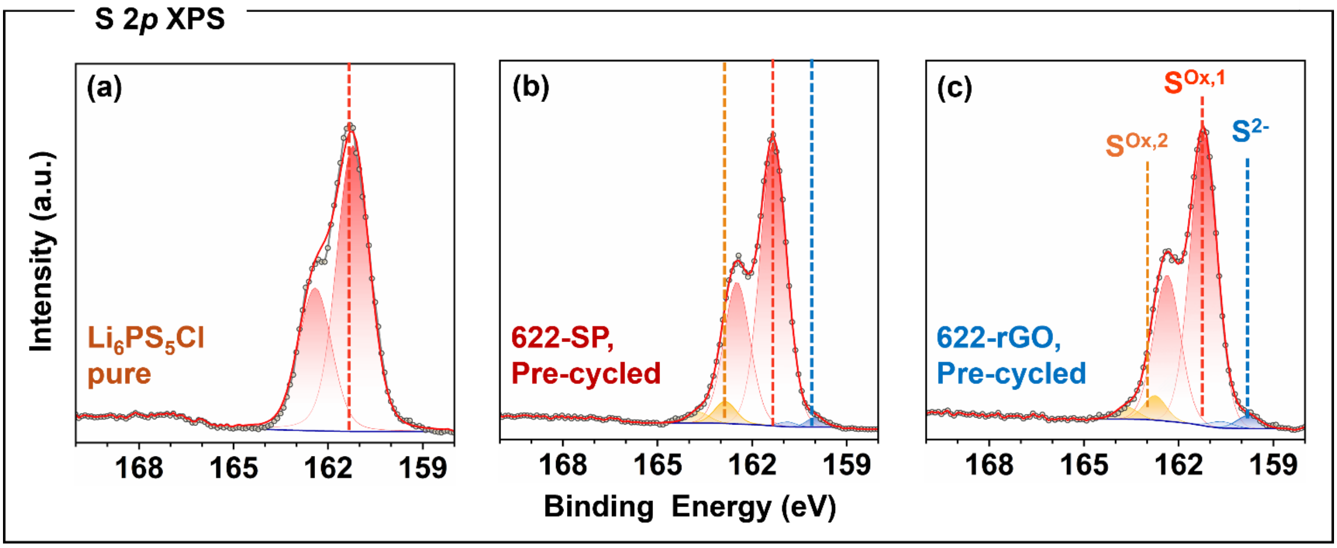

Figure S12. S 2*p* region XPS data of (a) pure Li_6PS_5Cl, (b) NMC 622-SP-Li_6PS_5Cl composite before cycling, and (c) NMC 622-rGO-Li_6PS_5Cl composite before cycling. In the fitted XPS spectra, black hollow circles represent experimental data, the red solid line denotes the overall fitted data, and the shaded regions stand for fitted individual components as indicated in (c) for the S 2*p* region. For detailed description of different components refer to the main manuscript.

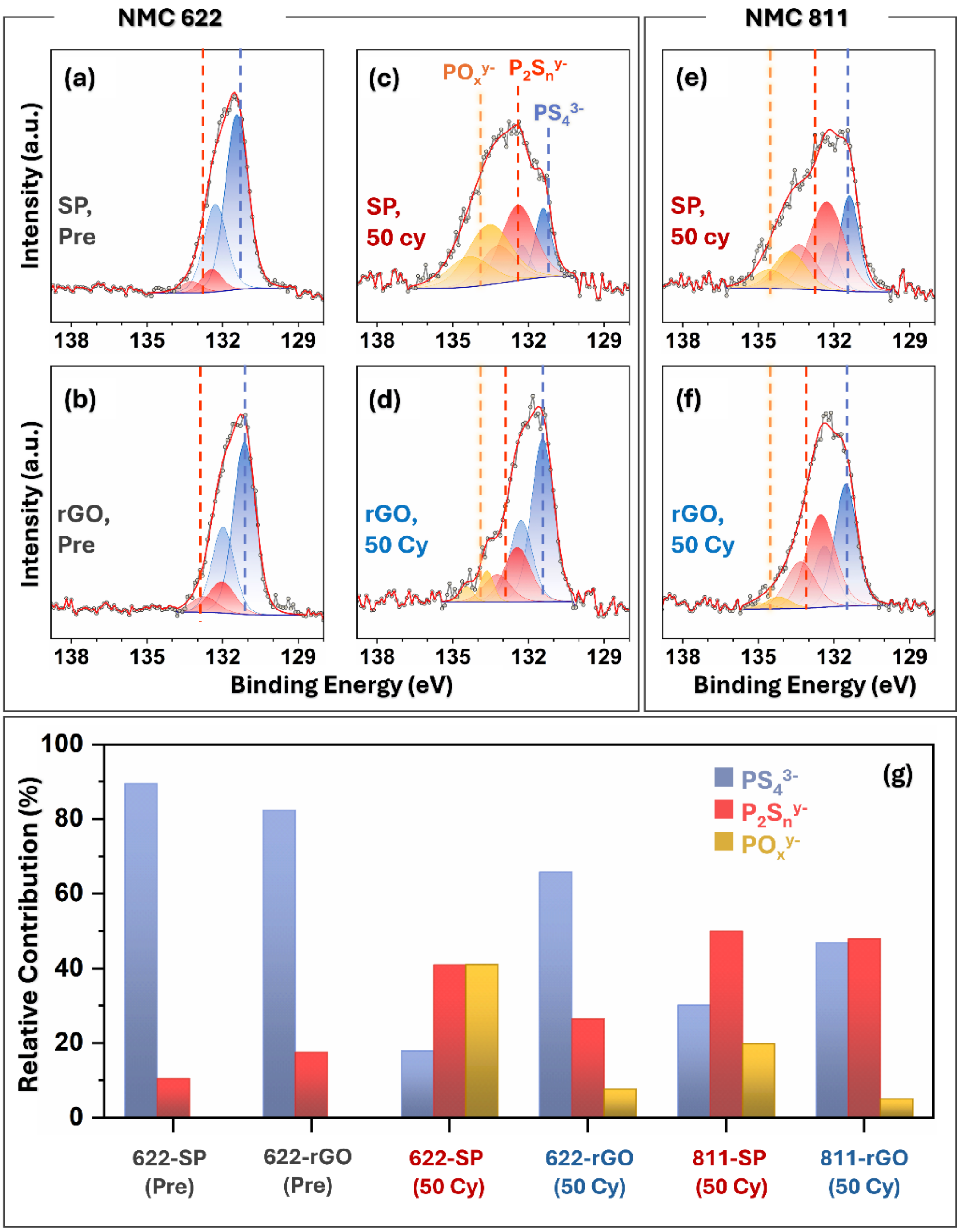

Figure S13. X-ray photoelectron spectroscopy (XPS) investigation of the cathode interfacial evolution. P 2*p* region XPS spectra for (a, b) NMC622 cathode before cycling (or pre cycling), nearly identical for both (a) SP and (b) rGO based cathode, and for NMC622 cathode with (c) SP and (d) rGO as the conductive carbon after 50 cycles. S 2*p* region XPS spectra for NMC811 cathode with (e) SP and (f) rGO after 50 cycles. (G) The relative contribution of the S 2*p* components, corresponding to different sulfur-containing species in the CEI, inferred from the XPS data in (a-f). In the (a-f) fitted XPS data, grey hollow circles represent experimental data, the red solid line denotes the overall fitted data, and the shaded regions stand for fitted individual components as indicated in (b)

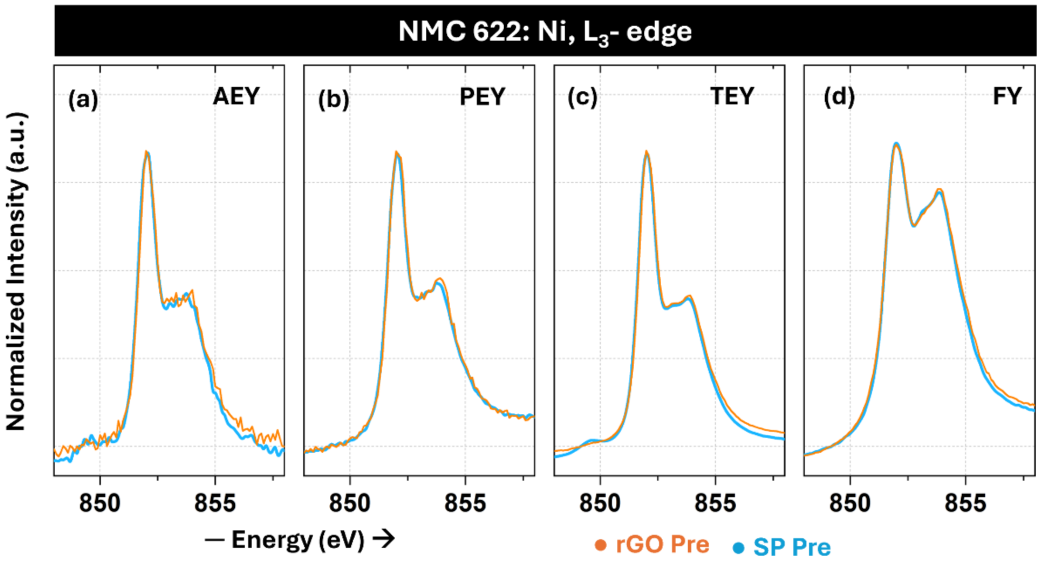

Figure S14. NEXAFS Ni L3-edge spectra acquired in (a) AEY, (b) PEY, (c) TEY, and (d) FY mode for the NMC622 cathode composites before cycling. Both rGO (orange) and SP-based (sky-blue) composites show identical Ni, L3-edge signatures on different collection modes, showing a uniform distribution of Ni oxidation states from the surface to the subsurface layer.

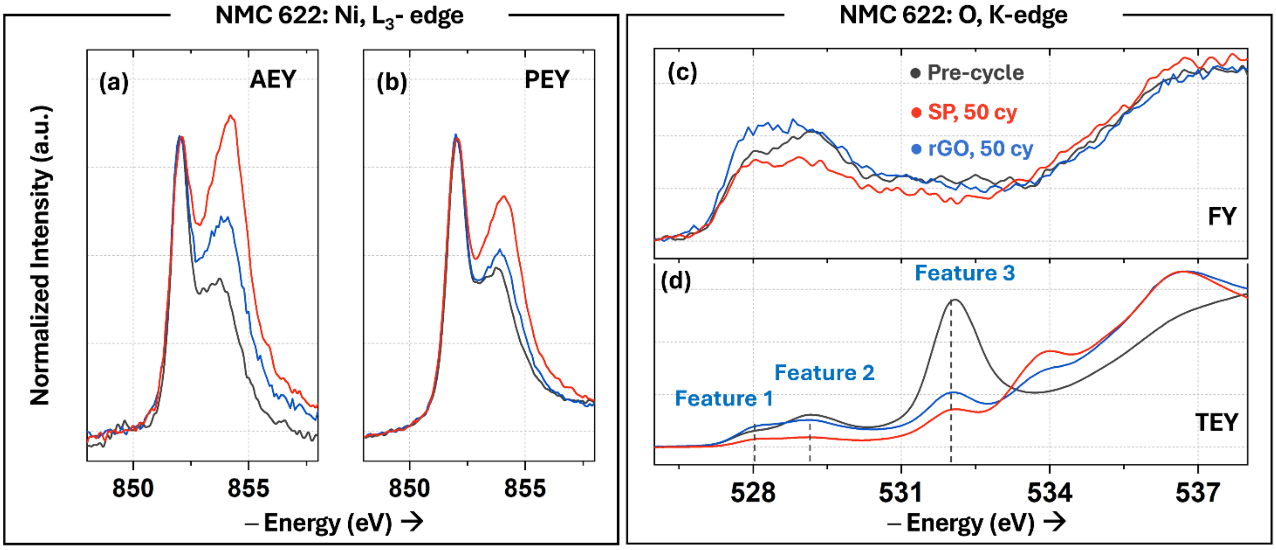

Figure S15. NEXAFS investigation of the cathode interface. (a) AEY and (b) PEY mode data show comparative Ni L3-edge features for NMC622 cathode with SP and rGO as the conductive cathode additive in pre- and post-cycled conditions. O K-edge features for the same electrodes in (c) FY and (d) TEY mode.

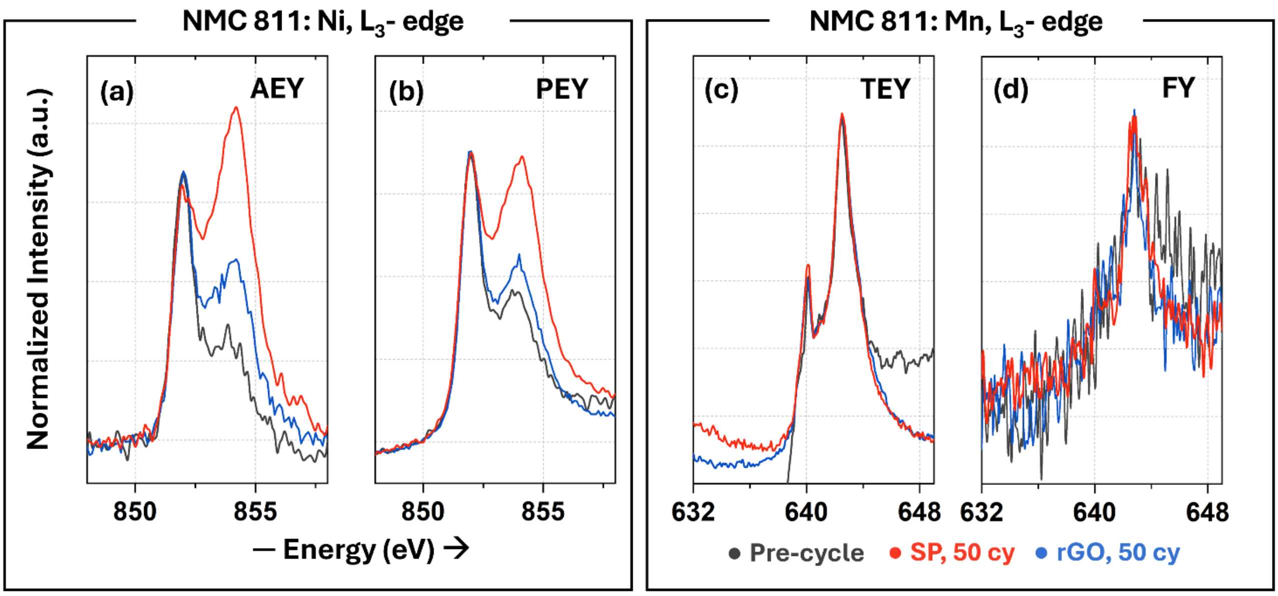

Figure S16. NEXAFS investigation of the cathode surface. (a) AEY and (b) PEY mode data show comparative Ni L_3-edge features for NMC811 cathode with SP and rGO as the conductive cathode additive in pre- and post-cycled conditions. Mn L_3-edge features for the same cells show no variation in (c) TEY and (d) FY modes.

Table S4. 2nd cycle discharge capacities (mAh g^{-1}) of NMC811 and NMC622 with SP and rGO at 60°C across various C rates, showing pre and post-100 cycles at 1C performance.

Cell	2nd cycle capacity (mAh g^{-1})								1C (100 cycles)
	0.3 C		0.5 C	1 C	2 C	3 C	4 C	5 C	
NMC 811-SP	Pre-100 cycles	155	139	121	116	113	111	107	**114 (1st)**
	post-100 cycles	111	55	25	20	19	18	18	**68 (100th)**
NMC 811-rGO	Pre	170	158	137	122	116	111	107	**133 (1st)**
	Post	136	123	104	93	86	81	77	**112 (100th)**
NMC 622-SP	Pre	183	137	92	62	35	23	18	**78 (1st)**
	Post	64	36	15	6	3.5	2	1.5	**33 (100th)**
NMC 622-rGO	Pre	177	167	151	140	135	131	128	**154 (1st)**
	Post	160	145	127	117	113	110	108	**140 (100th)**

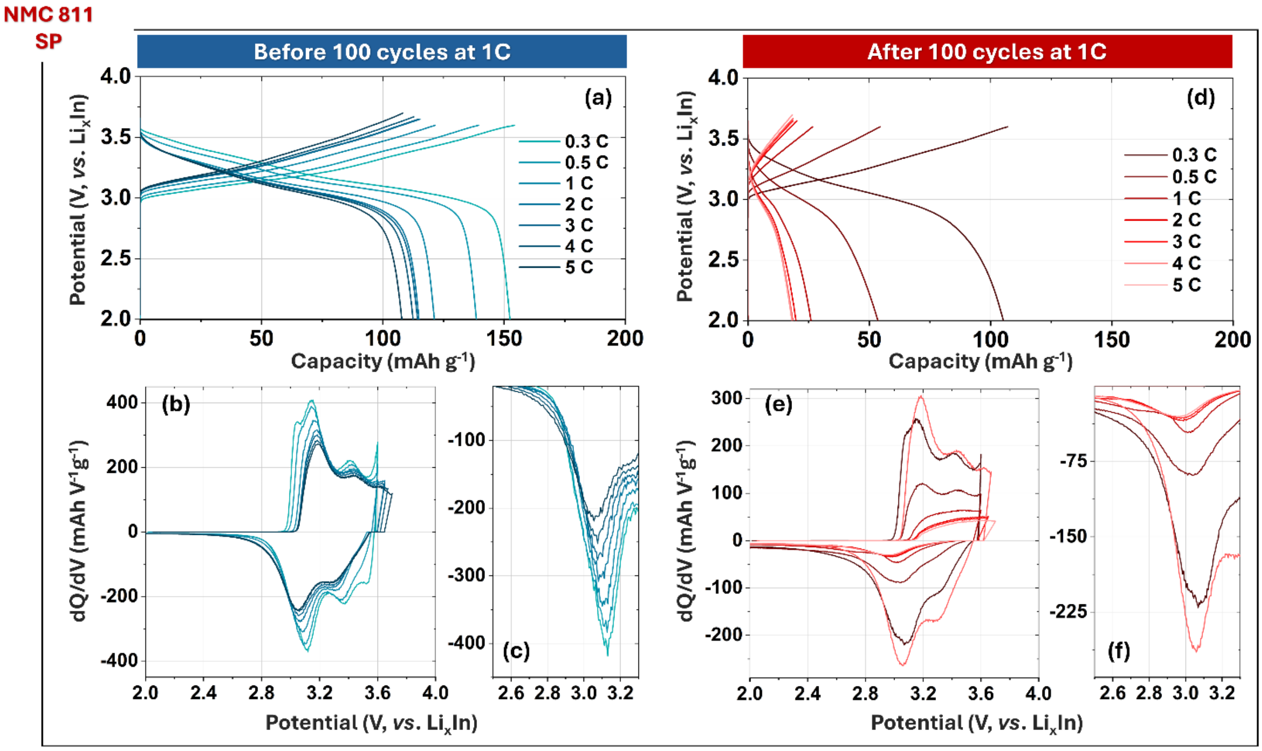

Figure S17. Comparison of NMC 811-SP ASSB cell rate capability data pre- and post-100 cycles at the 1C rate. (a) The galvanostatic polarization profile for the second cycle at each current rate, with (b, c) showing the corresponding dQ/dV plots before 100-cycle aging. (d) Galvanostatic polarization profile for the second cycle at each current rate, with (e, f) showing the corresponding dQ/dV plots post the 100-cycle aging. The testing was conducted at 60°C.

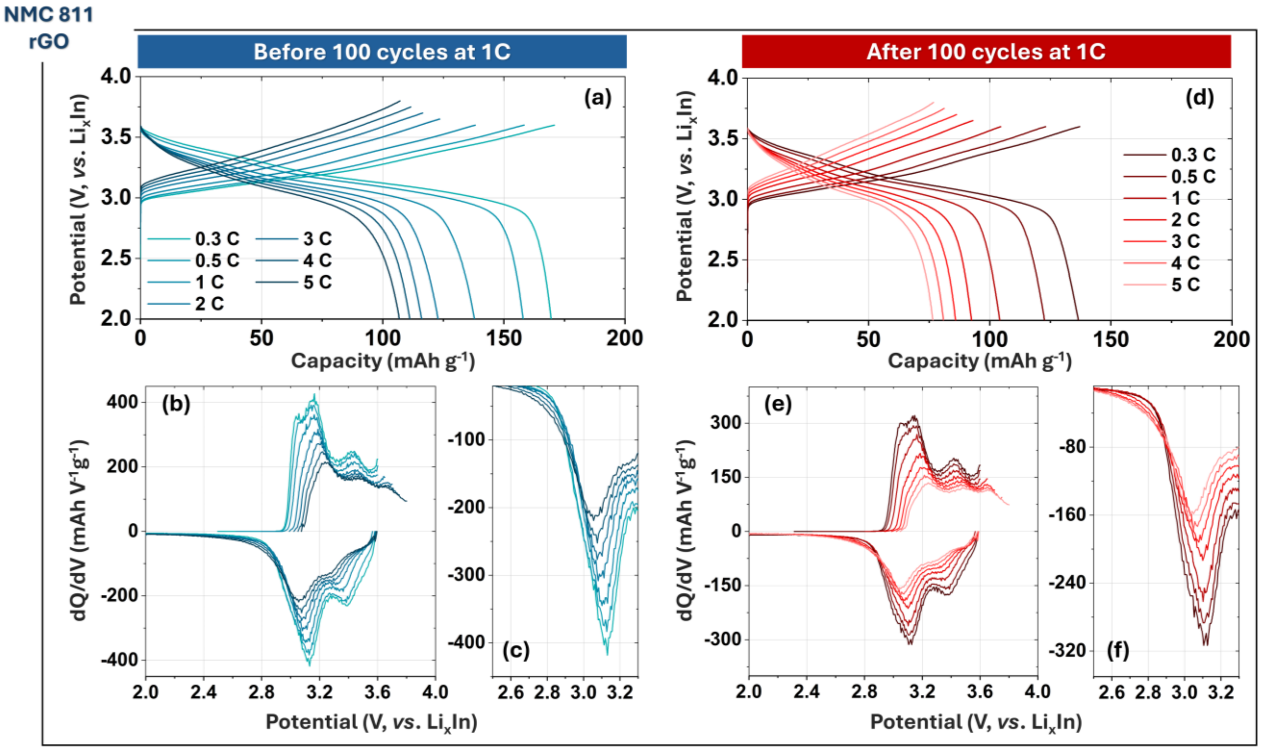

Figure S18. Comparison of NMC 811-rGO ASSB cell rate capability data pre- and post-100 cycles at the 1C rate. (a) The galvanostatic polarization profile for the second cycle at each current rate, with (b, c) showing the corresponding dQ/dV plots before 100-cycle aging. (d) Galvanostatic polarization profile for the second cycle at each current rate, with (e, f) showing the corresponding dQ/dV plots post the 100-cycle aging. The testing was conducted at 60°C.

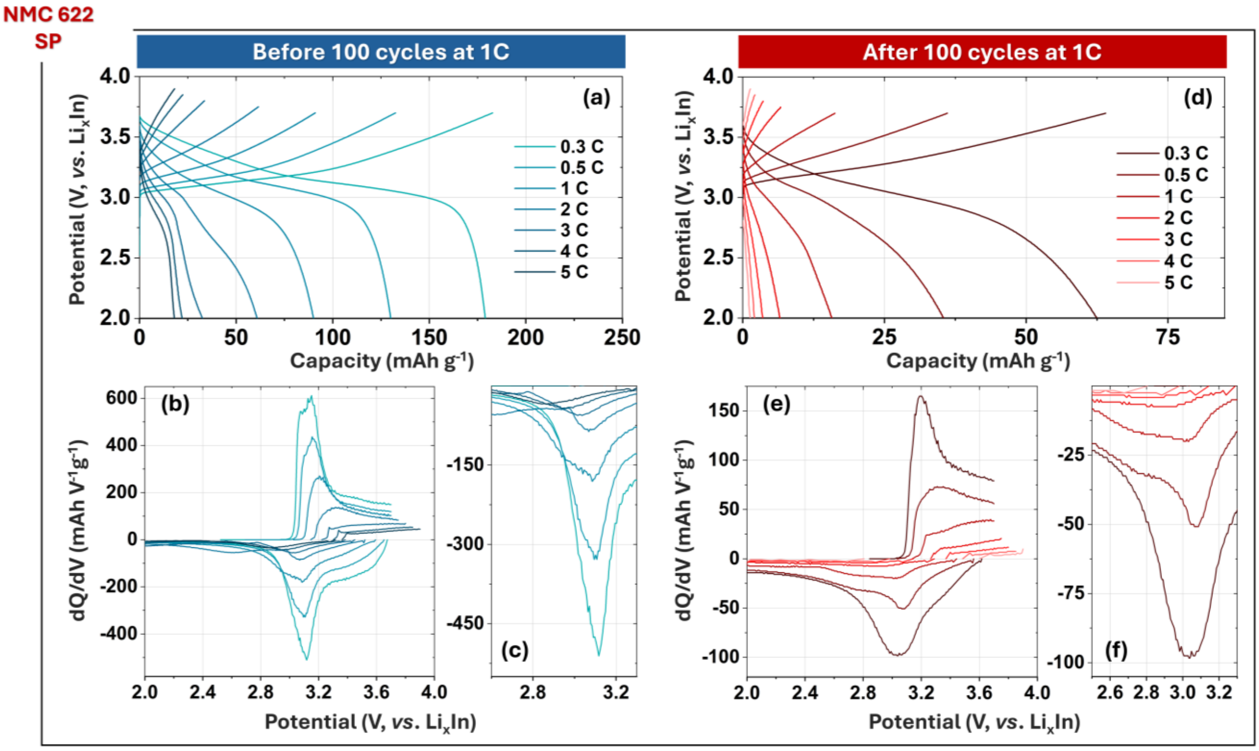

Figure S19. Comparison of NMC622-SP ASSB cell rate capability data pre- and post-100 cycles at the 1C rate. (a) The galvanostatic polarization profile for the second cycle at each current rate, with (b, c) showing the corresponding dQ/dV plots before 100-cycle aging. (d) Galvanostatic polarization profile for the second cycle at each current rate, with (e, f) showing the corresponding dQ/dV plots post the 100-cycle aging. The testing was conducted at 60°C.

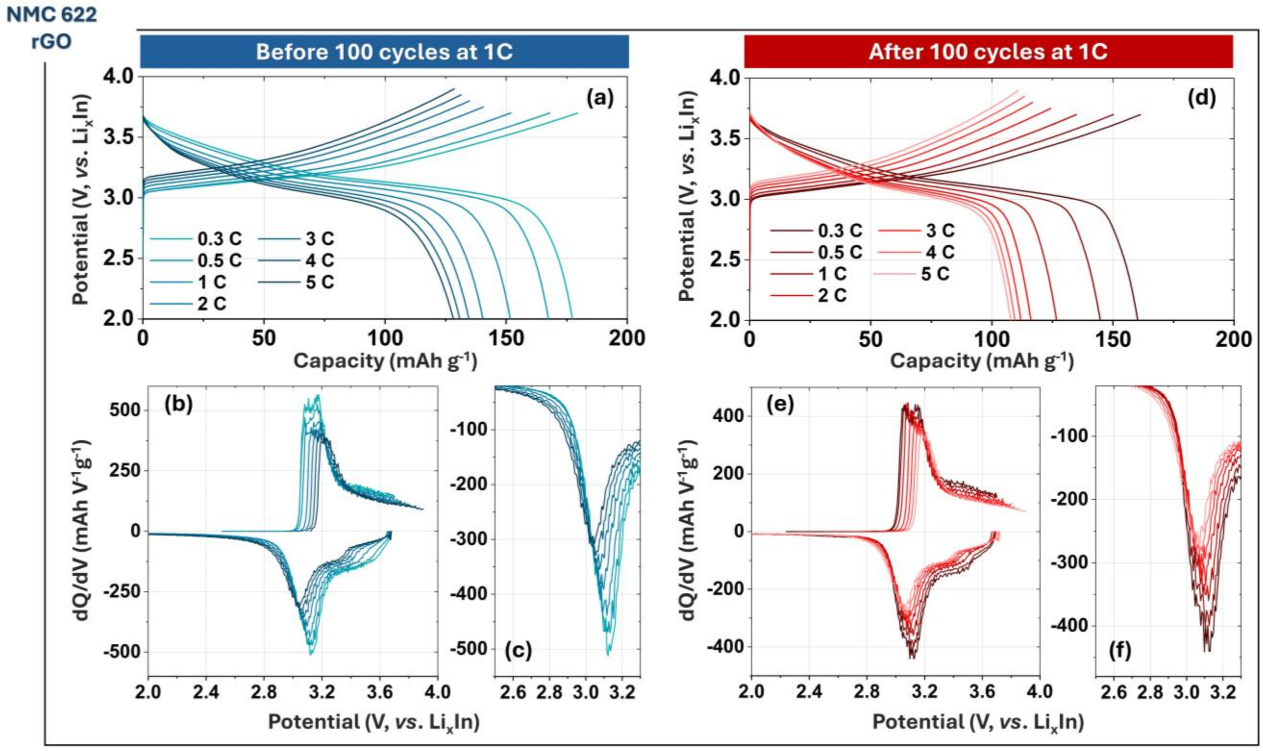

Figure S20. Comparison of NMC622-rGO ASSB cell rate capability data pre- and post-100 cycles at the 1C rate. (a) The galvanostatic polarization profile for the second cycle at each current rate, with (b, c) showing the corresponding dQ/dV plots before 100-cycle aging. (d) Galvanostatic polarization profile for the second cycle at each current rate, with (e, f) showing the corresponding dQ/dV plots post the 100-cycle aging. The testing was conducted at 60°C.

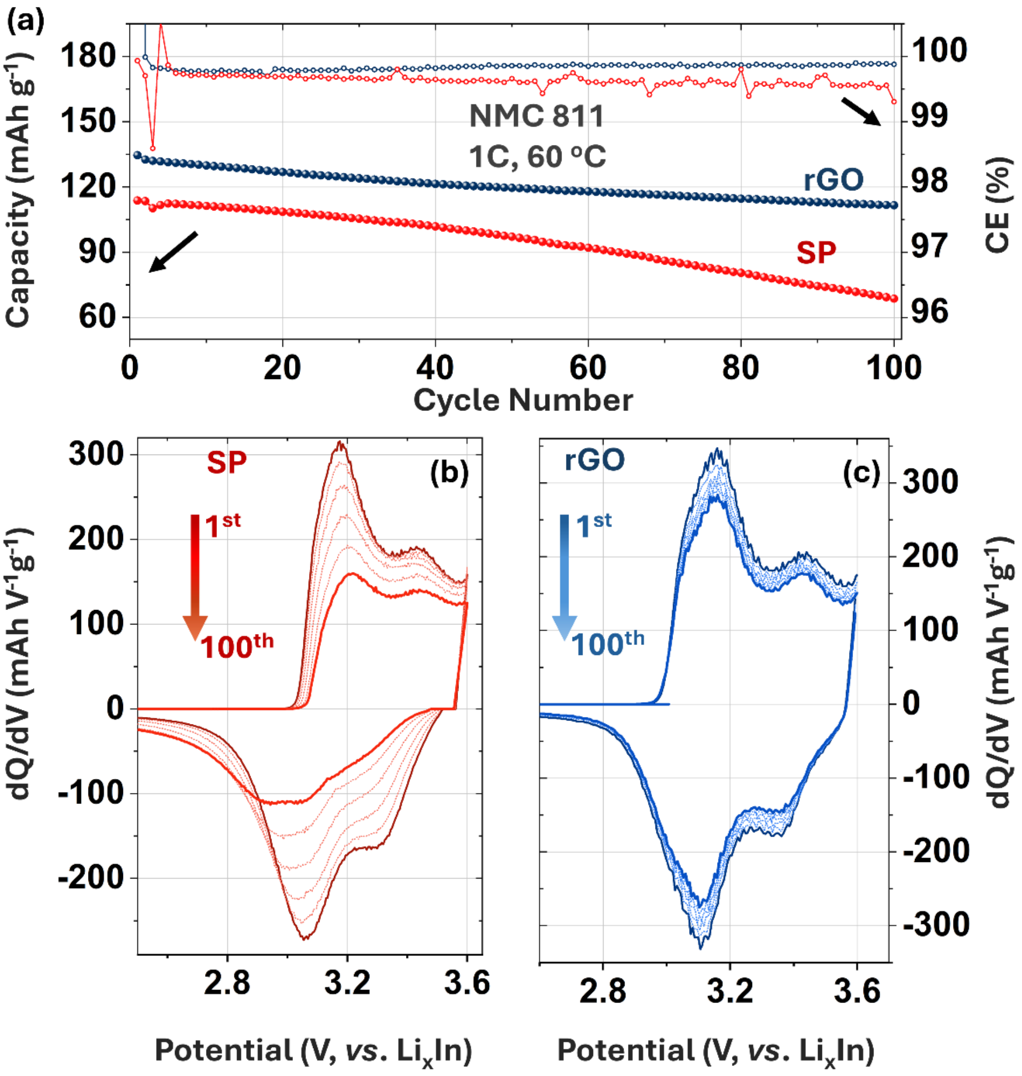

Figure S21. (a) Galvanostatic cycling performance of NMC811 ASSB cells with rGO and SP as conductive cathode additive at a 1C rate (between the rate-capability test), where 1C= 160 mAh g^{-1}, in 2.0-3.6 V (*vs.* $Li_{0.5}In$) window at 60 ºC with an active NMC loading of ~12 mg cm^{-2}. The evolution of the differential capacity profile as a function of cycling for the (b) SP and (c) rGO based cathode.

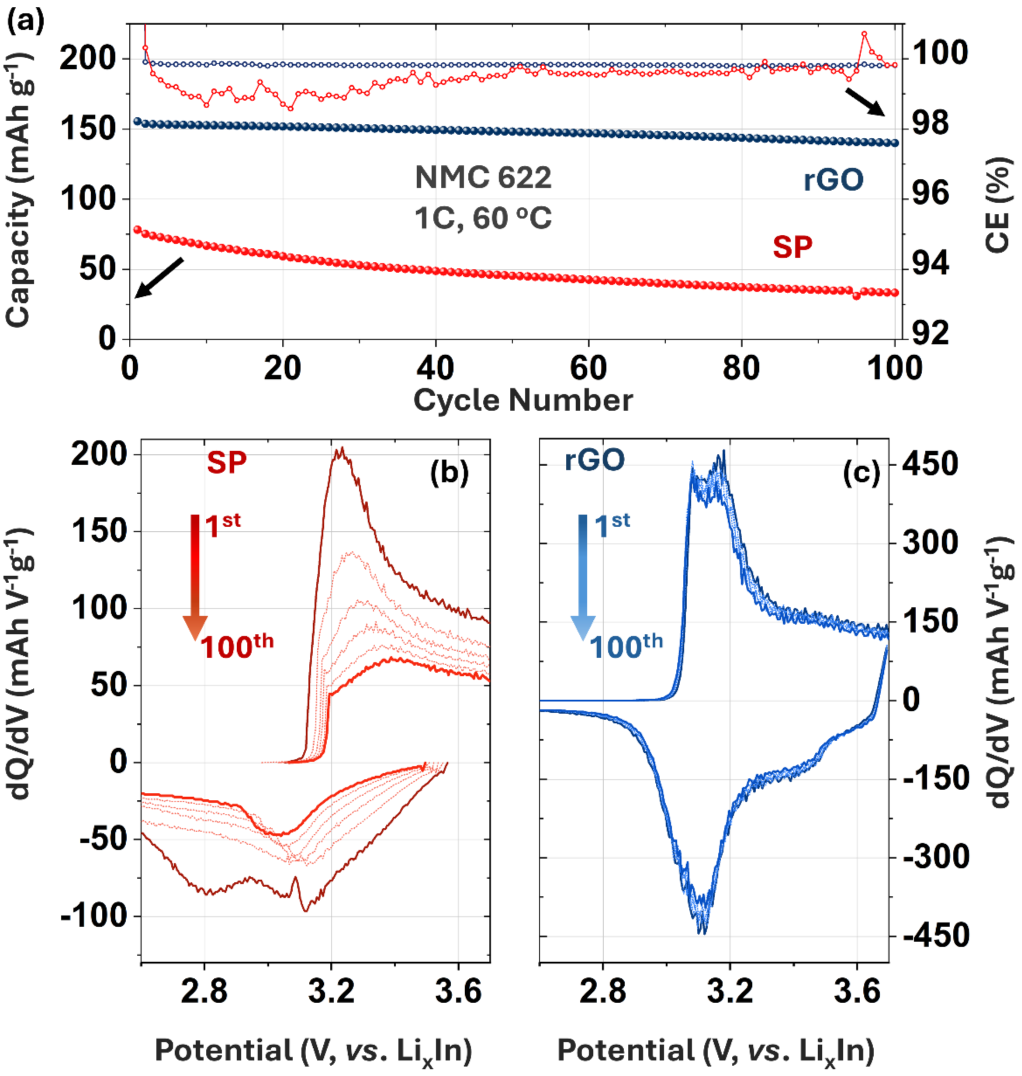

Figure S22. (a) Galvanostatic cycling performance of NMC622 ASSB cells with rGO and SP as conductive cathode additive at a 1C rate (between the rate-capability test), where 1C= 160 mAh g^{-1}, in 2.0-3.7 V (*vs*. $Li_{0.5}In$) window at 60 ºC with an active NMC loading of ~12 mg cm^{-2}. The evolution of the differential capacity profile as a function of cycling for the (b) SP and (c) rGO-based cathode.

Reference:

[1] T. H. Wan, M. Saccoccio, C. Chen, F. Ciucci, *Electrochimica Acta* **2015**, *184*, 483-499. https://doi.org/https://doi.org/10.1016/j.electacta.2015.09.097.

[2] A. Maradesa, B. Py, T. H. Wan, M. B. Effat, F. Ciucci, *Journal of The Electrochemical Society* **2023**, *170*, 030502. https://doi.org/10.1149/1945-7111/acbca4.

[3] M. Saccoccio, T. H. Wan, C. Chen, F. Ciucci, *Electrochimica Acta* **2014**, *147*, 470-482. https://doi.org/https://doi.org/10.1016/j.electacta.2014.09.058.

[4] A. Orue Mendizabal, M. Cheddadi, A. Tron, A. Beutl, P. López-Aranguren, *ACS Applied Energy Materials* **2023**, *6*, 11030-11042. https://doi.org/10.1021/acsaem.3c01894.

[5] J. O. Bonsu, A. Bhadra, D. Kundu, *Advanced Science n/a*, 2403208. https://doi.org/https://doi.org/10.1002/advs.202403208.
[6] A. Bhadra, M. Brunisholz, J. O. Bonsu, D. Kundu, *Advanced Energy Materials* **2025**, *15*, 2403608. https://doi.org/https://doi.org/10.1002/aenm.202403608.
[7] B. C. C. Cowie, A. Tadich, L. Thomsen, *AIP Conference Proceedings* **2010**, *1234*, 307-310. https://doi.org/10.1063/1.3463197.
[8] Y. Zhang, J. A. Alarco, J. Y. Nerkar, A. S. Best, G. A. Snook, P. C. Talbot, B. C. C. Cowie, *ACS Applied Energy Materials* **2020**, *3*, 2856-2866. https://doi.org/10.1021/acsaem.9b02489.
[9] E. Gann, C. R. McNeill, A. Tadich, B. C. C. Cowie, L. Thomsen, *Journal of Synchrotron Radiation* **2016**, *23*, 374-380. https://doi.org/doi:10.1107/S1600577515018688.
